# Mitochondrial dysfunction generates aggregates that resist lysosomal degradation in human breast cancer cells

**DOI:** 10.1038/s41419-020-2658-y

**Published:** 2020-06-15

**Authors:** Thomas G. Biel, Baikuntha Aryal, Michael H. Gerber, Josè G. Trevino, Naoko Mizuno, V. Ashutosh Rao

**Affiliations:** 1 Laboratory of Applied Biochemistry, Division of Biotechnology Review and Research III, Office of Biotechnology Products, Center for Drug Evaluation and Research, Food and Drug Administration, Silver Spring, MD 20993 USA; 2 Department of Surgery, College of Medicine, University of Florida, Gainesville, FL 32611 USA; 3 Cellular and Membrane Trafficking, Max Planck Institute of Biochemistry, Am Klopferspitz 18, Martinsried, Germany

**Keywords:** Protein aggregation, Mitophagy

## Abstract

Disrupting functional protein homeostasis is an established therapeutic strategy for certain tumors. Ongoing studies are evaluating autophagy inhibition for overcoming chemotherapeutic resistance to such therapies by neutralizing lysosomal pH. New and sensitive methods to monitor autophagy in patients are needed to improve trial design and interpretation. We report that mitochondrial-damaged breast cancer cells and rat breast tumors accumulate p53-positive protein aggregates that resist lysosomal degradation. These aggregates were localized to enzymatically-active autolysosomes that were degrading autophagosomes and the autophagic receptor proteins TAX1BP1 and NDP52. NDP52 was identified to associate with aggregated proteins and knocking down NDP52 led to the accumulation of protein aggregates. TAX1BP1 was identified to partly localize with aggregates, and knocking down TAX1BP1 enhanced aggregate formation, suppressed autophagy, impaired NDP52 autophagic degradation and induced cell death. We propose that quantifying aggregates and autophagic receptors are two potential methods to evaluate autophagy and lysosomal degradation, as confirmed using primary human tumor samples. Collectively, this report establishes protein aggregates and autophagy receptors, TAX1BP1 and NDP52, as potential endpoints for monitoring autophagy during drug development and clinical studies.

## Introduction

Inhibition of proteasome function is a standard treatment component for certain cancers like multiple myeloma^1^, but has limited activity in patients with metastatic breast cancer^2^. Autophagy is a proposed mechanism that contributes to proteasome-inhibitor drug resistance^3,4^. However, previous clinical studies using hydroxychloroquine as an adjuvant treatment for different cancer types have provided conflicting results regarding autophagy inhibition and antitumor activity^5^. New methods to accurately characterize autophagy in patients is a critical gap in this field and limits the interpretation of such trials^6^. Here, we investigate protein aggregates and the autophagic mechanism to identify mechanistically-relevant targets and methods that may improve the clinical assessment of autophagy.

Dysfunctional autophagy is a collective term for any impairment in the autophagic process that prevents the degradation of the autophagic cargo^7^. Selective autophagy is the recognition of specific cargo, such as protein aggregates (aggrephagy) and dysfunctional mitochondria (mitophagy)^8^. Autophagic receptor proteins facilitate selective cargo recognition by utilizing ubiquitin binding domains to recognize poly-ubiquitinated misfolded proteins and a LC3 binding domain to associate with the autophagosome^9,10^. Several mammalian autophagic receptors are known to recognize poly-ubiquitinated proteins on the mitochondria for mitophagy^11^, but the ability to recognize aggregated proteins independent of the mitochondria remains unclear. Clinical trials have heavily focused on p62, a selective autophagic receptor protein, accumulation as a biomarker to demonstrate autophagy inhibition. However, the results for p62 accumulation in the presence of hydroxychloroquine are not consistent between studies^12,13^. Additional autophagic receptor proteins have demonstrated to undergo lysosomal degradation in non-small cell lung cancer cell lines^14^, and are highly expressed in the several cancer cell lines including breast (MDA-MB-231, SKBR3, and MCF-7)^15,16^. However, the autophagic roles of these receptor proteins in cancer cells have not been characterized.

Mitochondrial dysfunction in cancer cells can promote protein aggregate formation, mitophagy, malignant transformation, and cell death^17^. Mitochondrially-targeted redox agents (MTAs) induce mitochondrial dysfunction and autophagy, selectively in cancer cells^18,19^. In this report, we monitored the formation and degradation of protein aggregates in cancer cells with dysfunctional mitochondria using two MTAs: Mitoquinone (MitoQ) and Mitoapocynin (MitoApo). Dysfunctional mitochondria caused an accumulation in p53 positive protein aggregates that resist lysosomal degradation leading to cell death. In contrast to p62, TAX1BP1 and NDP52 were identified as two autophagic receptor proteins being degraded by autophagy. These findings that TAX1BP1 and NDP52 are degraded but p53 positive protein aggregates accumulate in cancer cells may provide the opportunity to develop methods to assess for functional autophagy with defective aggrephagy, which could serve as a more pragmatic biomarker for characterizing autophagy during early clinical drug development.

## Results

### Protein aggregates are sequestered by autophagosomes in breast cancer cell lines with dysfunctional mitochondria

**Fig. 1:**
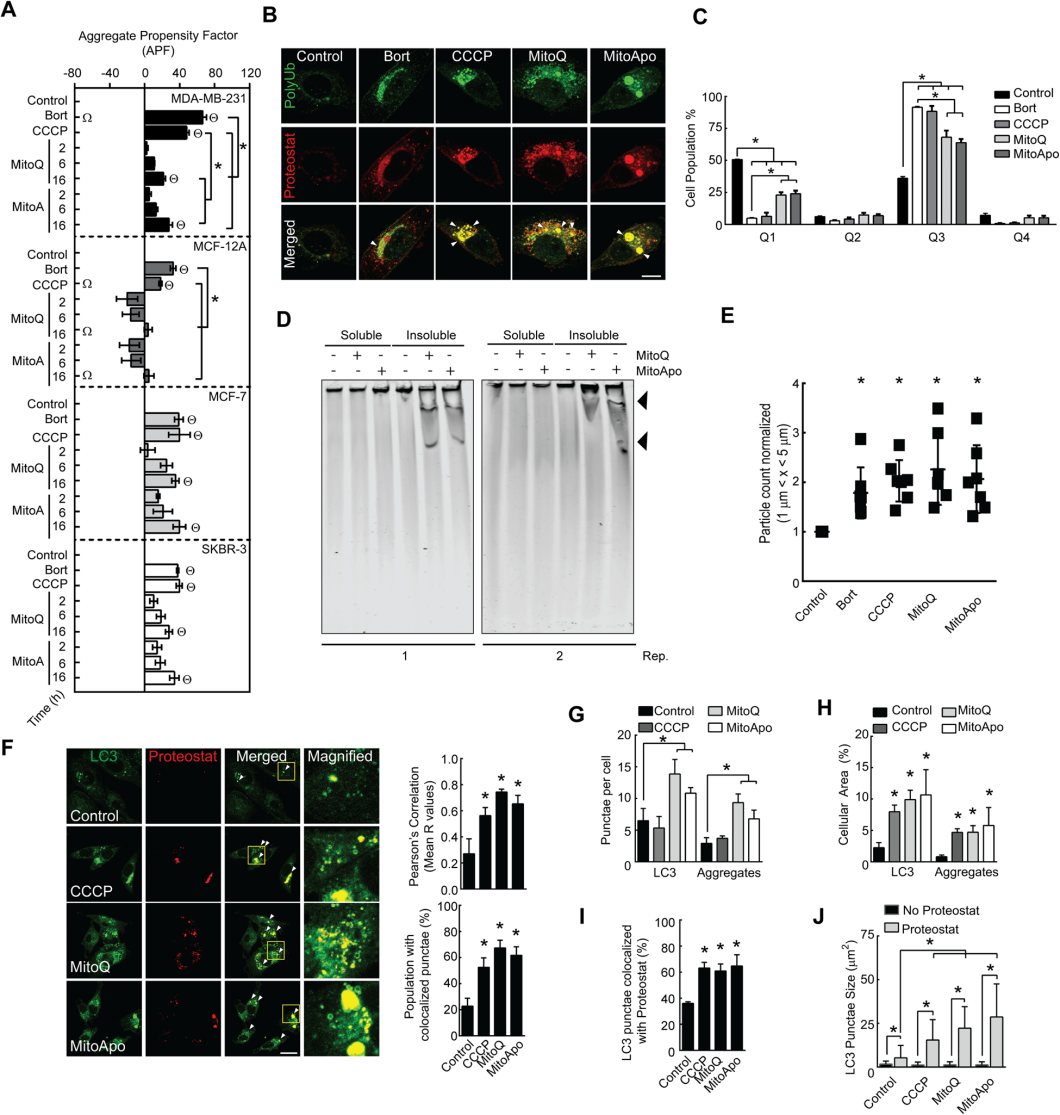
MTAs induced the formation of protein aggregation that co-localize with autophagosomes in breast cancer cell lines. **a** MDA-MB-231, MCF-12A, MCF-7, and SKBR-3 cells were exposed to DMSO (control), Bortezomib (Bort) (5 nM) and CCCP (30 μM) for 16 h or MitoQ (1 μM) and MitoApo (1 μM) for 2, 6, and 16 h to determine the aggregation propensity factor (APF) using FACS analysis. Bars represents the mean and error bars are SEM. (Two-way ANOVA using 16-h results, *n* = 3–5, *ΘΩ indicate *p* > 0.05 as according to Tukey’s post-hoc test. Θ is a significant difference to the cell type control, * is significant difference between the treatments within the cell type, and Ω is a significant difference between all the cell types for the indicated treatment. **b** Representative immunofluorescent images of poly-ubiquitination (Green) and aggregates (Red) of MDA-MB-231 cells treated with DMSO, Bort, CCCP, MitoQ, or MitoApo for 16 h. Arrow heads indicate areas of co-localization. Scale Bar is 10 μm. **c** Quantification of from K-63 immune-labeled and Proteostat-stained MDA-MB-231 cells scatter plots from FACS analysis. Scatter plots were subdivided into quadrants to assess protein aggregate formation (Proteostat) and K-63 poly-ubiquitination in MDA-MB-231 cells subjected to the indicated treatments for 16-h. Bars represents the mean and error bars are SEM. (Two-way ANOVA, *n* = 4, *p > 0.05 as according to Tukey’s post-hoc test.) **d** Soluble and insoluble fractions from MDA-MB-231 cells exposed to DMSO, MitoQ, or MitoApo for 48 h underwent native agarose-acrylamide gel composition electrophoresis. Two representative gels stained with Coomassie brilliant blue are shown. Black arrow head identified large (>450 kDa) insoluble protein bands in MitoQ and MitoApo treated MDA-MB-231 cells. **e** Particle quantification of the MDA-MB-231 cell lysate after DMSO, Bort, CCCP, MitoQ, or MitoApo exposure for 48 h using microflow imaging. Dots represent individual experiments and error bars indicate SEM (ANOVA, *n* = 7, *p > 0.05 as according to Tukey’s post-hoc test.) **f** Fluorescent images of LC3 and Proteostat punctae in MDA-MB-231 cells after 24-h exposure to DMSO, CCCP MitoQ, or MitoApo. White arrow heads and yellow boxes indicate areas of colocalization and magnification, respectively. Scale bars are 20 μΜ. Graphs show the Pearson’s R values using whole cells, and cell population percentage with at least 1 colocalized punctae. (*n* = 4–7 fields (5 × 5 tiles)). **g**–**h** Quantification of LC3 and Proteostat (**g**) punctae per cell and (**h**) area stained within the cells (*n* = 4–7 fields, *p < 0.05 as indicated by Tukey’s post-hoc test as compared to the respective stain control cells). **i** Percentage of Proteostat-labeled LC3 punctae within the cells (*n* = 4–8 fields). **j** Areal dimensions of LC3 punctae with and without Proteostat (*n* = 350 LC3 punctae per treatment).

Mitochondrial dysfunction has been linked to the formation and accumulation of misfolded protein aggregates^20^. To investigate MTA induced protein aggregation, the aggregation propensity factor was compared between four different breast cell lines (three cancerous, and one non-cancerous) exposed to MitoQ and MitoApo. Similar to previous reports^21–23^, Bortezomib, a proteasome inhibitor, and carbonyl cyanide m-chlorophenyl hydrazine (CCCP), a mitochondrial uncoupling agent, induced protein aggregation as indicated by an increase in the aggregation propensity factor (Fig. 1a and Supplementary Fig. 1a). Protein aggregates in the MTA-treated breast cancer cell lines did increase as compared to the non-treated cancer cells or the non-cancerous cell line (Fig. 1a) and prolonging the treatment resulted in further aggregate accumulation (Supplementary Fig. 1b). This data suggests that acute MTA treatment induced the onset of protein aggregation selectively in breast cancer cell lines as compared to the non-cancerous MCF-12A cells.

Aggregated proteins that contain lysine-63 (K-63) linked poly-ubiquitin chains are transported by autophagosomes to the lysosome for degradation^10,24,25^. MTA-treated cells did contain Proteostat-stained aggregates that colocalized with K-63 ubiquitin chains (Fig. 1b) and scatter plot analyses revealed that the entire cell population had increased K-63-labeled and Proteostat-stained proteins in cells following Bortezomib, CCCP, and MTA treatments (Fig. 1c, and Supplementary Fig. 1c). To further characterize these protein aggregates, soluble and insoluble protein fractions were subjected to composition native electrophoresis (CNG)^26^ and micro-flow imaging (MFI)^27^. CNG gels revealed that large (>450 kDa) protein bands were only detectable in the insoluble fractions from MTA-treated cells (Fig. 1d) and MFI demonstrated a quantifiable increase in sub-visible particles 1–5 μm in size within cells subjected to Bortezomib, CCCP, and MTAs (Fig. 1e). These data confirm that MTAs cause acute dysfunctional proteostasis in cancer cells leading to the formation of large insoluble protein aggregates that are ubiquitinated with K-63 linkages.

MitoQ and MitoApo induce autophagy in MDA-MB-231 cells^18^. To investigate MTA-induced aggrephagy, MDA-MB-231 with dysfunctional mitochondria were Proteostat-stained and LC3-immunolabeled to determine autophagosomal recognition of protein aggregation (Supplementary Table 1). Each of the MTAs caused a significant increase in Proteostat and LC3-II punctae colocalization (Fig. 1f) and the number of Proteostat colocalized LC3 punctae per cell (Fig. 1g). Overlapping fluorescent areas of LC3 punctae and LAMP1 indicated autophagosome/lysosomal fusion as previously described^28,29^. This correlation was applied to fluorescent areas of LC3 with Proteostat punctae to indicate autophagosomes containing protein aggregates. Spatial measurements for LC3 and Proteostat punctae in cells with and without mitochondrial dysfunction indicate that MTAs increase the cellular occupancy of LC3 and Proteostat punctae (Fig. 1h and Supplementary Table 1) and the number of cells with colocalized punctae (Fig. 1i). To characterize the size of the potential autophagosomes that contained aggregates, LC3 punctae with and without overlapping Proteostat were measured to reveal that mitochondrial damage did increase the size of the Proteostat-labeled LC3 punctae (Fig. 1j). In contrast to the MTA treatment, CCCP-treated cells did not increase in the number of punctae per cell (Fig. 1g), but the size (Fig. 1h), % area coverage (1H), and number of the Proteostat-labeled LC3 punctae (Fig. 1i) were comparable between CCCP-treated and MTA-treated cells. One potential speculation for these results is that CCCP-induced aggregates, as compared to MTAs, may have fewer aggregate structures with a greater density of misfolded protein, which is supported by the differential incorporation of the rotor dye. (Fig. 1a).These data suggest that MTAs induce the formation of K63 poly-ubiquitinated protein aggregates that are sequestrated by autophagosomes.

### Mitochondrial dysfunction induced the formation of p53 positive aggregates that resist lysosomal degradation

**Fig. 2:**
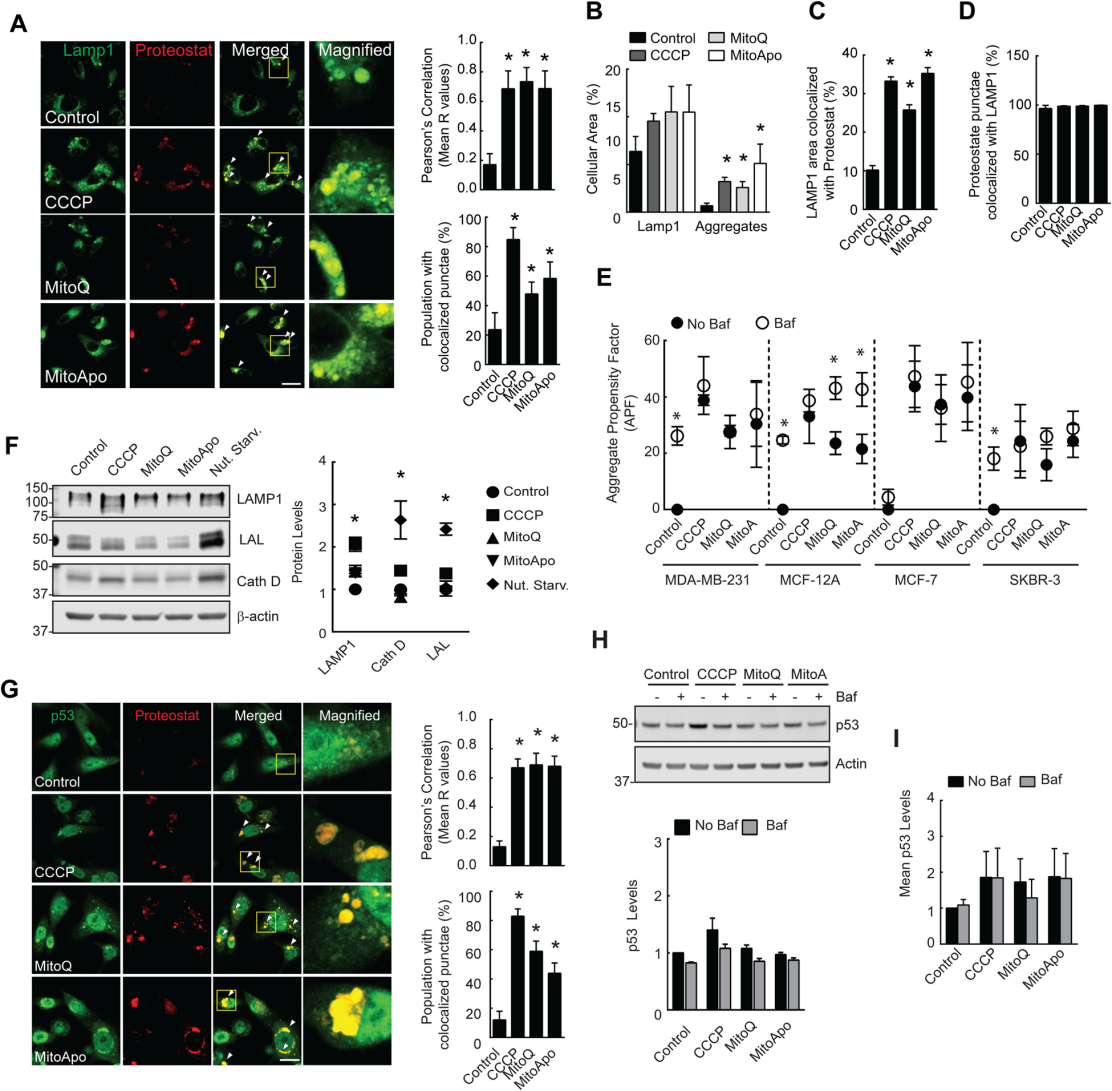
Mitochondrial dysfunction generated p53 positive aggregates that resist lysosomal degradation. **a** Fluorescent images of LAMP1 and Proteostat punctae in MDA-MB-231 cells exposed to mitochondrial damaging agents. Graphs show the Pearson’s R values. (*n* = 4–8 fields) (**b**–**d**) Quantification of the (**b**) LAMP1 or Proteostat stained areas, (**c**) LAMP1 stained area with Proteostat and (**d**) Proteostat stained area with LAMP1 overlapping. (*n* = 4–8 fields, *p < 0.05 as indicated by Tukey’s post-hoc test as compared to the respective stain control cells). (**e**) Cells were exposed to DMSO, CCCP, MitoQ, and MitoApo in the presence or absence of Bafilomycin (5 nM) for 24 h to determine the APF using FACS analysis. (*n* = 4–5). **f** Immunoblots of LAMP1, Cath D, and LAL from MDA-MB-231 cells after 24 h of nutrient starvation or treatment (*n* = *7*). **g** Immunofluorescent images of p53 in Proteostat-stained MDA-MB-231 cells subjected to CCCP, MitoQ and MitoApo for 24 h. Graphs are the Pearson’s Correlation (R values shown) of whole cells and the population percentage positive for colocalized punctae. (ANOVA, *n* = 3–7 fields, *p < 0.05 as indicated by Tukey’s post-hoc test as compared to the control). **h** Representative immunoblot of p53 from MDA-MB-231 cells after 24 h of CCCP, MitoQ, and MitoApo treatment in the presence and absence of Baf (*n* = *3*, *p < 0.05 as indicated by Tukey’s post-hoc test as compared to the control) (**i**) Mean p53 fluorescence in cells treated with different mitochondrially targeted agents for 24 h in the presence and absence of Baf. (*n* = *3*) using FACS. Bars represents the mean and error bars are standard deviation. Bars and dots represent the mean, while error bars are standard deviation. *p > 0.05 as according to Tukey’s post-hoc test.

Autophagosomes fuse with the lysosome to degrade their luminal content. To demonstrate aggregates within the autophagosomes were transported to the lysosome, colocalization studies were performed between lysosomal associated membrane protein 1 (LAMP1) and Proteostat. Mitochondrial dysfunction increased LAMP1 colocalization with Proteostat punctae, and the percentage of cells with colocalized punctae (Fig. 2a, and Supplementary Fig. 2). Similar to a previous report^30^, the LAMP1 cellular area did modestly increase in cells with dysfunctional mitochondria (Fig. 2b). To further characterize the quantity of lysosomes with aggregates, spatial measurements of the LAMP1 stained areas with and without Proteostat were quantified (Fig. 2c). Mitochondrial dysfunction did increase Proteostat punctae colocalization with LAMP1 by ~20% suggesting that a portion of lysosomes harbor protein aggregates. More importantly, Proteostat punctae were not exclusively detected without LAMP1 (Fig. 2d). With >99% Proteostat punctae associating with lysosomes, it would be reasonable to speculate that the LC3 punctae colocalized with Proteostat punctae (Fig. 1i) had been delivered to the lysosome.

To determine lysosomal dependent aggregate degradation, cells were exposed to mitochondrial damaging agents in the presence and absence of Balifomycin, a lysosomal neutralizing agent, to monitor for changes in the aggregate levels (Fig. 2e). Bafilomycin-treated MDA-MB-231, MCF-12A, and SKBR-3 cell lines, but not MCF-7 cells, had a similar increase in the aggregate content as compared to non-treated cells. MTA-induced mitochondrial dysfunction caused an increase in protein aggregation in all the cell lines, but only the non-cancerous MCF-12A cell line demonstrated aggrephagic flux. To support that MitoQ impaired the degradation of aggregates in MDA-MB-231 cells, the lysosomal luminal content was evaluated using electron micrographs. Single membrane bound vesicles, likely representing autolysosomes^29^ were observed to contain undigested mitochondria and filamentous aggregate like structures^31,32^ (Supplementary Fig. 2a). These data support that MTAs selectively cause dysfunctional aggrephagy by impairing lysosomal degradation in breast cancer cell lines as compared to the non-cancerous cell line.

To investigate whether protein aggregate accumulation was independent of autophagosomal maturation, wild type and autophagy-deficient ATG7 knock-out MEFs were treated with Bortezomib and MitoQ to monitor for changes in protein aggregation (Supplementary Fig. 2b). Non-stressed MEF cells with deficient autophagosomal maturation had an elevated aggregation propensity factor as compared to wild-type cells (Supplementary Fig. 2c, d), which supports the presence of functional aggrephagy in WT MEF cells. However, Bortezomib and MitoQ treatment did not further increase the aggregate content in autophagy defective cells as compared to the wild type control (Supplementary Fig. 2b–d). Thus, defects in autophagosomal generation did not enhance the accumulation of protein aggregates in Bortezomib or MitoQ treated cells. These data demonstrate that mitochondrial damage can impair lysosomal degradation of aggregates in cell with or without functional autophagosomal maturation.

CCCP-induced mitochondrial dysfunction has demonstrated to impair lysosomal function^30^. To characterize MTA-induced lysosomal dysfunction, the lysosomal acidity, vesicular structure, and enzyme levels were characterized. MTA treatment caused a kinetic increase in vesicular lysotracker red sequestration (Supplementary Fig. 2e) and the lysosomal vesicles appeared to be larger in MitoQ-treated cells as compared to control cells (Supplementary Fig. 2f). To determine if lysosomal biogenesis may have a potential role in lysosomal impairment, three lysosomal enzymes were quantified in cells with and without mitochondrial dysfunction and nutrient starvation, a known inducer of lysosomal biogenesis^33^. In contrast to the increase in lysosomal size and quantity, a corresponding increase in lysosomal enzymes was absent (Fig. 2f) in cells treated with MTAs. These data demonstrate that autophagosomes sequester and transport aggregates to the lysosome, but lysosomal degradation is impaired leading to aggregate accumulation.

To test a well-characterized protein that may accumulate due to mitochondrial dysfunction, p53 was investigated because genetic point mutations are known to increase the protein’s propensity to aggregate under low pH conditions^34^, mitochondrial dysfunction causes cytosolic acidosis^35^, amyloid-like protein aggregates co-localize with p53 in human breast cancer biopsies and MDA-MB-231 cells^34,36,37^, and preclinical studies have demonstrated that p53-positive aggregates resist degradation and contribute to chemotherapeutic resistance^38^. To demonstrate that mitochondrial dysfunction contributes to the formation of lysosomal resistant protein aggregates, p53 colocalization with Proteostat-marked aggregates was determined in MDA-MB-231 cells (Fig. 2g and Supplementary Table 3). In the presence of mitochondrial dysfunction, a clear association between protein aggregates and p53 was detected, but the protein levels of p53 were shown to be consistent with or without lysosomal neutralization (Fig. 2h, i). These data suggest that mitochondrial dysfunction induced by MTAs lead to the accumulation of lysosomal degradation resistant p53 positive aggregates in MDA-MB-231 cells.

### NDP52 was selectively degraded by the lysosome and recognized protein aggregates

Protein aggregates are known to be recognized by autophagic receptor proteins but the subsequent degradation and turnover of these proteins remain unsolved and controversial. p62 is a well-known autophagy receptor that recognizes ubiquitinated aggregated proteins to facilitate autophagic degradation, but the degradation has been reported to be suppressed in cells with mitochondrial damage^8,24,30^. To identify autophagic receptors that accumulate or degrade during mitochondrial damage-induced lysosome impairment, the stability and degradation of the autophagic receptor proteins were investigated in MTA-treated MDA-MB-231 cells. Among the different autophagic receptors investigated, NDP52 was the only autophagic receptor that decreased in cells treated with cycloheximide (CHX), a protein synthesis inhibitor (Supplementary Fig. 3a). To determine if MTAs activated the degradation of these autophagic receptors, CHX-exposed cells were subjected to MTA treatment to monitor for changes in the protein quantities. In contrast to OPTN and p62, MTAs induced TAX1BP1 loss and a change in the protein’s half-life from >24 h to ~6.4 h.

**Fig. 3:**
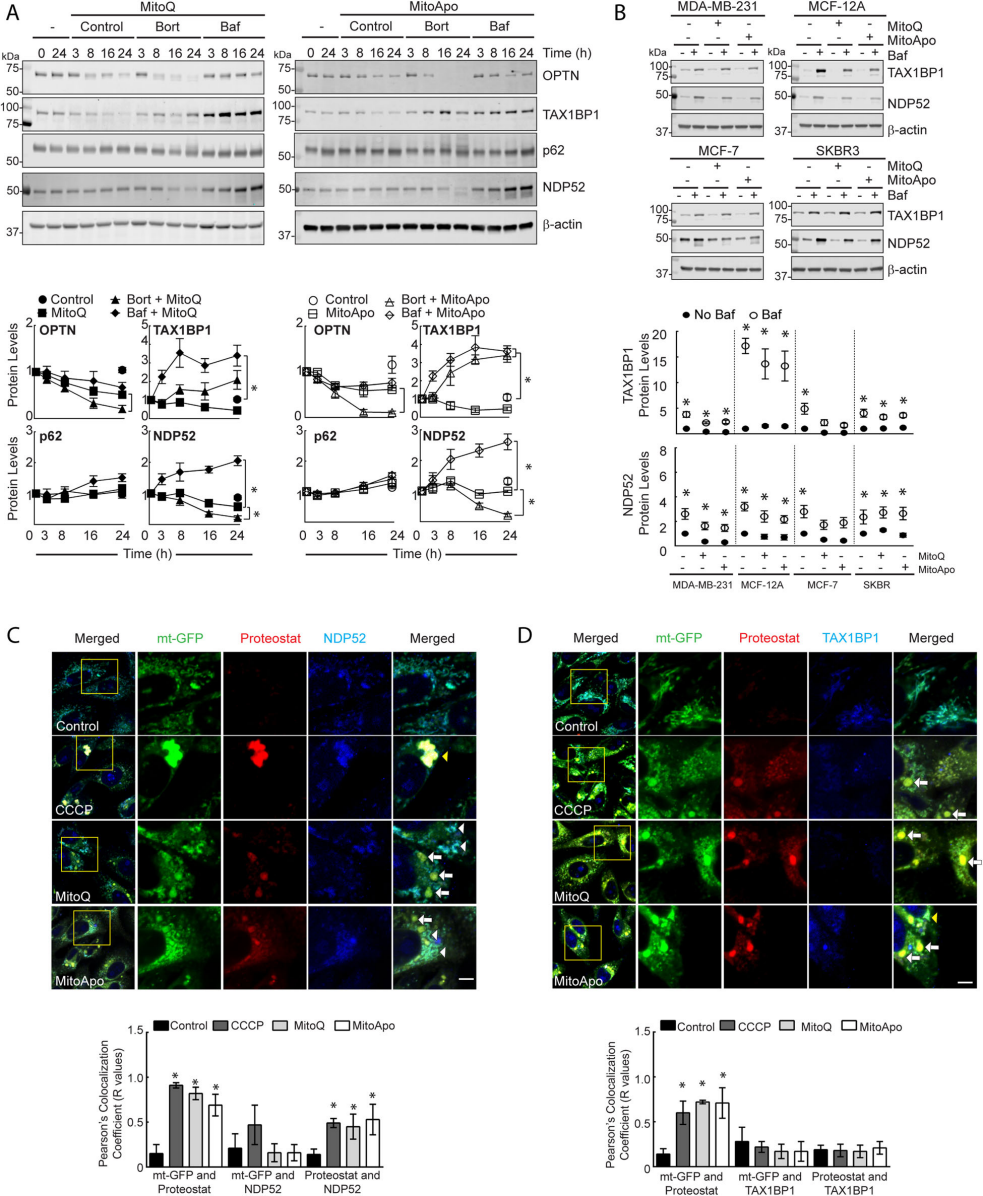
TAX1BP1 and NDP52 are selectively degraded by the lysosome following MTA treatment, but only NDP52 delivers aggregated protein to the lysosome. **a** Representative immunoblots of the OPTN, TAX1BP1, p62, and NDP52 from MDA-MB-231 cells exposed to MitoQ (1 μM) or MitoApo (1 μM) in the presence or absence of Bortezomib (Bort) (5 nM) or Baf (5 nM) at the indicated times (ANOVA at 24 h per protein, *n* = 3–5, *p < 0.05 as according to Tukey’s post-hoc test between treatments, while #*p* < 0.05 compared to all treatments.) **b** Representative immunoblots of TAX1BP1 and NDP52 in MDA-MB-231, MCF-12A, MCF-7, and SKBR3 cells exposed to MitoQ (1 μM) or MitoApo (1 μM) in the presence or absence of Baf (5 nM) for 24 h (two-way ANOVA per cell line for MTA dependency on Baf effects, *n* = 3–5, *p < 0.05 as according to Tukey’s post-hoc test at 24-h. two-way ANOVA for cell type dependency on treatment effects, #*p* < 0.05 indicates difference between cell types per treatment) (**c**) TAX1BP1 and (**d**) NDP52 immuno-labeled mt-GFP expressing MDA-MB-231 cells stained with Proteostat. Cells were treated with DMSO (Control), CCCP (30 μM), MitoQ (1 μM) or MitoApo (1 μM) for 24 h. Scale bar is 5 μm. Whole cells were used to establish the Pearson’s correlation coefficient (*R* values shown) between mt-GFP, autophagic receptor, and Proteostat (ANOVA per comparison, *n* = 3–8 *fields* (5 × 5 *tiles per field*), *p < 0.05 according to Tukey’s post-hoc test identified significant differences between the control and indicated treatment.). For all graphs, the dots represent mean and error bars are SEM.

To identify the degradation mechanism contributing to the loss of TAX1BP1 or NDP52, the proteasome or autophagy pathway was inhibited, and the protein levels were quantified. MTA treatment with lysosomal neutralization lead to an increase in TAX1BP1 and NDP52, but not OPTN or p62 (Fig. 3a). NDP52 degradation was primarily through the lysosome, while TAX1BP1 degradation was both lysosomal and proteasomal. LC3-II is proposed to recognize these adapters^11^, and co-immunoprecipitation confirmed that TAX1BP1 and NDP52 were in a protein complex with LC3-II (Supplementary Fig. 3b). These data suggest that NDP52 and TAX1BP1 are selectively degraded by the lysosome in MTA-treated cells with dysfunctional aggrephagy.

To expand these findings, the lysosomal degradation of TAX1BP1 and NDP52 were compared between the breast cell lines with and without MTA treatment. All the cell lines in the absence or presence of mitochondrial dysfunction had an increase in the NDP52 and TAX1BP1 levels following the Bafilomycin treatment (Fig. 3b and Supplementary Fig. 3c). However, the accumulation of TAX1BP1 in the MCF12A cell line as compared to the cancerous cell lines had a greater fold change, which suggests that the TAX1BP1 degradation rate was greater this non-cancerous cell line.

TAX1BP1, NDP52, and p62 recognize poly-ubiquitinated proteins to facilitate mitophagy^11^. To determine if these autophagic receptors recognize aggregates or mitochondria, mt-GFP expressing MDA-MB-231 cells with dysfunctional mitochondria were immunolabeled and stained with Proteostat. In contrast to p53 that fully encompassed the aggregate-stained area (Fig. 2f), all the autophagic receptor punctae images demonstrated areas both with and without Proteostat colocalization (Fig. 3c, d, Supplementary Fig. 3d and 4, and Supplementary Tables 4–6). Consistently, the colocalization between mitochondria and Proteostat increased in cells with mitochondrial dysfunction. However, NDP52 was the only autophagic receptor to increase colocalization with Proteostat following mitochondrial dysfunction (Fig. 3c and Supplementary Table). These data suggest that NDP52 was recognizing the aggregated proteins, potentially facilitating the transport of the aggregate to the lysosome and was selectively degraded by the lysosome as compared to the aggregated proteins.

### TAX1BP1 mediates lysosomal degradation of NDP52 and autophagy flux

**Fig. 4:**
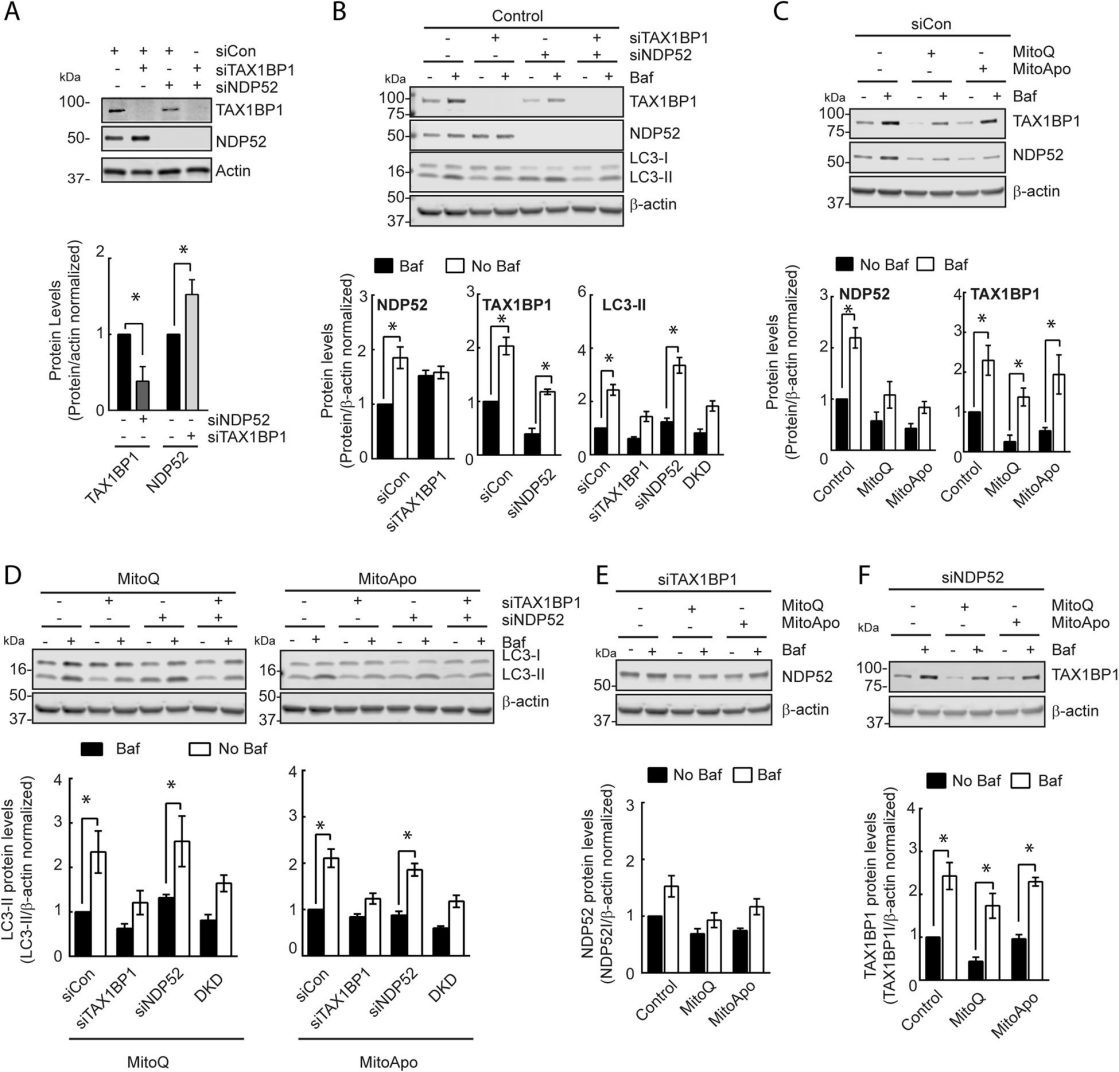
TAX1BP1 as an upstream regulator for the autophagic degradation of NDP52 and autophagic flux. **a** Representative TAX1BP1 and NDP52 immunoblots from MDA-MB-231 cells treated with siRNA for TAX1BP1, NDP52, or both (double knockdown (DKD)) (two-way ANOVA, *n* = *3*, *p < 0.05 according to Dunnett’s post-hoc test). **b** Immunoblots of TAX1BP1, NDP52, and LC3 from siCon-treated MDA-MB-231 cells in the presence and absence of Baf for 2 h. (two-way ANOVA, *n* = *3*, *p < 0.05 as indicated by Tukey’s post-hoc test for changes in LC3-II within the treatment groups) (**c**) Immunoblot of TAX1BP1 and NDP52 using control siRNA (siCon)-treated MDA-MB-231 cells subjected to a DMSO, MitoQ (1 μM) and MitoApo (1 μM) for 24 h and Baf (5 nM) for 2 h prior to protein harvest (two-way ANOVA per graph, *n* = 3–5, *p < 0.05 as indicated by Tukey’s comparison test) **d**) Immunoblots of LC3 using cells treated with siTAX1BP1, siNDP52, or in combination with or without MTA exposure in the presence or absence of Baf for 2 h prior to harvest. (two-way ANOVA, *n* = *3*, *p < 0.05 as indicated by Tukey’s post-hoc test for changes in LC3-II within the treatment groups) (**e**, **f**) Immunoblots of (**e**) NDP52 in siTAX1BP1-treated cells and (**e**) TAX1BP1 in siNDP52-treated cells in the presence and absence of MTA with and without Baf treatment for 2 h prior to harvest (two-way ANOVA per graph, *n* = 3–5, *p < 0.05 as indicated by Tukey’s comparison test) (Bars represent the mean and error bars are SEM).

To characterize the autophagic role of TAX1BP1 and NDP52 in MDA-MB-231 cells, knockdown studies were performed to assess the lysosomal turnover of NDP52, TAX1BP1, and LC3-II. Without any mitochondrial damaging agents, cells with TAX1BP1 knocked down did increase the levels of NDP52, while knock down of NDP52 decreased TAX1BP1 levels (Fig. 4a). To determine autophagic flux and lysosomal degradation in cells with TAX1BP1 or NDP52 knocked down, the levels of LC3-II, TAX1BP1, and NDP52 were measured and revealed that knocking down TAX1BP1 suppresses autophagic flux and the lysosomal degradation of NDP52 (Fig. 4b). In contrast, knocking down NDP52 did not affect TAX1BP1 degradation or autophagic flux, and double knock down did not further suppress autophagic flux (Fig. 4b). These data indicate that TAX1BP1 was an upstream regulator for the autophagic degradation of NDP52, and that the loss of TAX1BP1 suppressed autophagy.

To investigate autophagic flux and lysosomal turnover in cells with prolonged mitochondrial damage, MTAs were administered for 22 h followed by the addition of Bafilomycin for the final 2 h to assess the changes in NDP52, TAX1BP1, and LC3-II levels. Consistent with our previous results (Fig. 3b), scrambled siRNA-treated (SiCon) MDA-MB-231 cells exposed to MTAs demonstrated a loss of TAX1BP1 and NDP52 (Fig. 4c). The shorten duration of Bafilomycin did reveal that the lysosomal degradation of NDP52 was impaired in cells treated with MTAs (Fig. 4c), and that LC3-II was actively undergoing lysosomal degradation (Fig. 4d). However, additional knock down of TAX1BP1 in the presence of mitochondria dysfunction did lead to the suppression of autophagic flux (Fig. 4d) and the loss of NDP52 degradation (Fig. 4e). The loss of NDP52 in cells treated with MTA did not affect TAX1BP1 lysosomal turnover or autophagic flux with or without NDP52 downregulation (Fig. 4c, d, f). These data indicate that the extent of TAX1BP1 depletion in cells with dysfunctional mitochondria can exclusively regulate autophagic flux and lysosomal degradation of NDP52.

### NDP52 mediates intracellular protein aggregate content and mitophagy activation

**Fig. 5:**
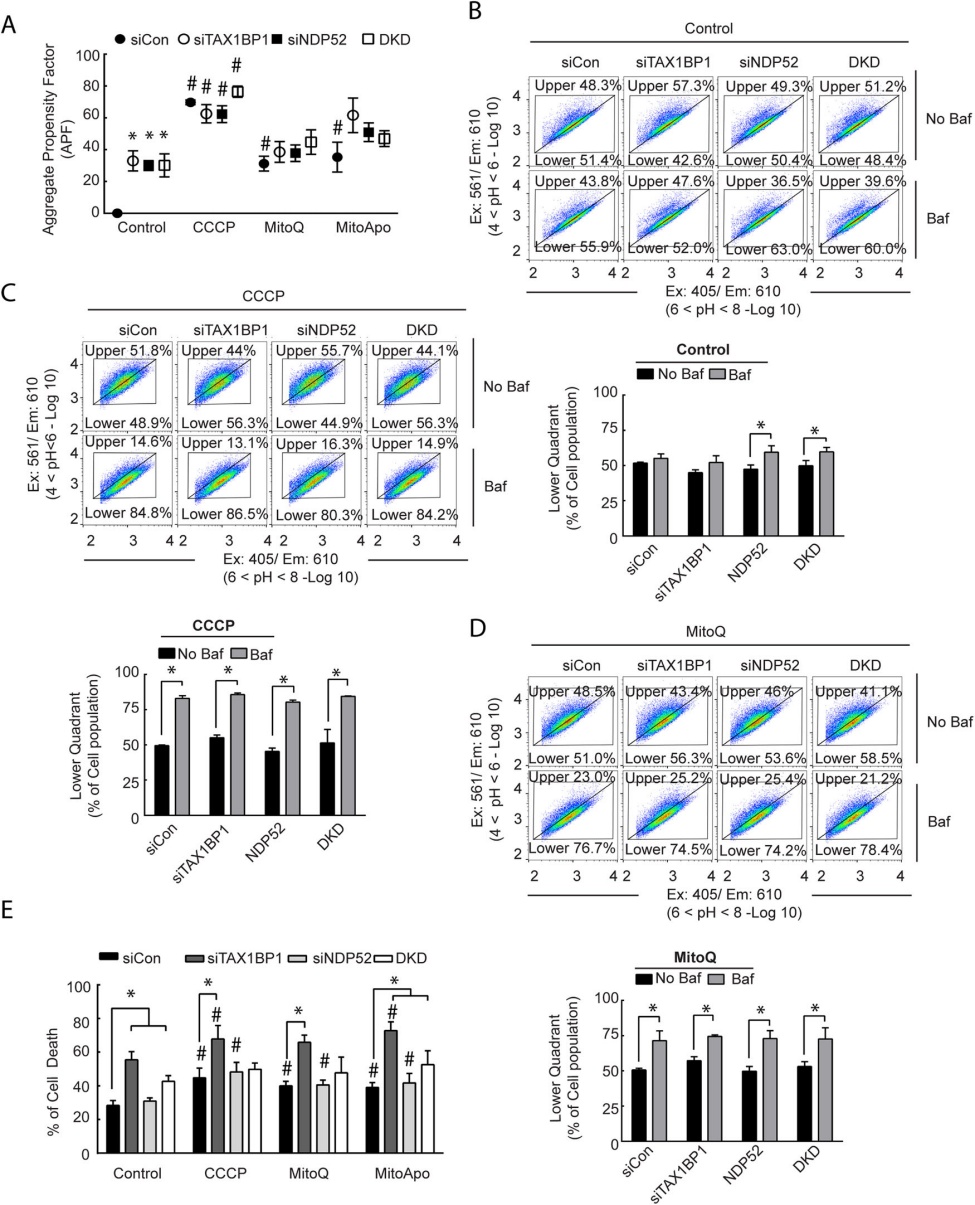
The loss of TAX1BP1 or NDP52 leads to the accumulation of protein aggregates in MDA-MB-231 cells, but the loss of TAX1BP1 was lethal. **a** FACS analysis of Proteostat in TAX1BP1 and NDP52 knockdown MDA-MB-231 cells treated with CCCP, MitoQ, and MitoApo for 24 h to determine the APF. (two-way ANOVA, *n* = *3*, *p < 0.05 as indicated by Tukey’s post-hoc test for changes in APF between the siRNA groups per treatment, while #p < 0.05 compares the indicated groups to the respective siRNA-treated cells exposed to the control treatment.) **b**–**d** Representative FACS divided scatter plot of TAX1BP1 and NDP52 knocked down MDA-MB-231 cells expressing mt-mKeima treated with (**b**) DMSO, (**c**) CCCP and (**d**) MitoQ treatment for 24 h in the presence and absence of Baf. (5 nM) for the last 2 h (Two-way ANOVA per treatment, *n* = 3, *p < 0.05 as indicated by Tukey’s post hoc test). **d** Quantification of Annexin V and propidium iodide (PI) stained MDA-MB-231 cells with TAX1BP1, and NDP52 knocked down following CCCP, MitoQ, and MitoApo treatments using FACS. Cell death values were determined by the sum Q1 (PI positive), Q2 (PI + annexin V positive), and Q4 (annexin V positive) (two-way ANOVA, *n* = *3*, *p < 0.05 as indicated by a Dunnett’s post-hoc test for comparing within a treatment group, while #*p* < 0.05 compares indicated groups to the respective siRNA-treated cells exposed to the control treatment.). Bars and dots represent the mean and error bars are standard deviation.

TAX1BP1 and NDP52 were identified as two autophagic receptor proteins undergoing lysosomal degradation in cells with dysfunctional mitochondria that accumulate protein aggregates. To determine if the loss of TAX1BP1 or NDP52 can exacerbate the accumulation of protein aggregates, TAX1BP1 and NDP52 knock down cells were treated with MTA to assess for protein aggregates (Fig. 5a). Knocking down TAX1BP1 and NDP52 did increase the aggregate content in cells without mitochondrial dysfunction as compared to the siCon-treated cells, which indicates that under basal conditions these autophagic receptor proteins are mediating the clearance of aggregates. However, the presence of MTA-induced mitochondrial dysfunction did not further increase aggregate content. CCCP was the only treatment that caused heightened protein aggregation in NDP52 and TAX1BP1 knocked-down cells as compared to the control. These data suggest that the loss of TAX1BP1 or NDP52 contribute to aggrephagy in MDA-MB-231 cells, but also that the loss of these receptors do not cause further protein aggregate accumulation in cells with dysfunctional mitochondria and impaired lysosomes.

MTAs induce the accumulation of mitochondrial PINK1 to activate mitophagy in MDA-MB-231 cells^18^. To expand on this finding in the context of autophagy receptors, the PINK1 levels were assessed in all the breast cell lines studied. Consistent with our previous report^18^, mitochondria dysfunction induced the accumulation of PINK1 in MDA-MB-231 cells (Supplementary Fig. 5a). However, PINK1 levels were not detectable in the other cell lines with or without mitochondrial dysfunction. These data indicate the PINK1 accumulation is an unreliable biomarker for protein aggregation.

To identify the role of NDP52 and TAX1BP1 in the delivery of mitochondria to the lysosome, cells expressing mt-mKeima were treated with siRNA followed by MTAs with or without Bafilomycin for the final 2 h. Fluorometric analysis of mt-mKeima utilizes a pH sensitive excitation shift, which indicates mt-mKeima translocation from an alkaline mitochondria matrix to acidic lysosomal lumen^11^. Control-treated cells with siTAX1BP1 did not activate mitochondrial delivery to the lysosome, but a modest increase was observed in NDP52− and NDP52+ TAX1BP1-knockdown cells (Fig. 5b). Consistent with our previous study^18^, MTAs activated mt-mKeima translocation to the lysosome and neutralizing the lysosome caused diminution of the acidic mt-mKeima signal (Fig. 5c, d). Cells with NDP52 and/or TAX1BP1 knocked down did not affect the delivery of mitochondria to the lysosome in the presence of MTAs (Fig. 5c, d). These data suggest that NDP52 and TAX1BP1 are not essential for translocating dysfunctional mitochondria to the lysosome in MDA-MB-231 cells.

To determine if the loss of TAX1BP1 or NDP52 impacted the viability of MDA-MB-231 cells in response to MTAs, deficient cells underwent annexin V and propidium iodide (PI) staining to quantify cell death (Supplementary Fig. 5b). Control-treated cells with TAX1BP1 or TAX1BP1 and NDP52 dual knocked down had a 20–27% increased in cells positive for both PI and annexin V as compared to the control or NDP52 knocked down cells (Fig. 5e). These data indicated that the loss of TAX1BP1 can induced cell death in MDA-MB-231 cells in a manner that is independent from NDP52. MTA-induced mitochondria damage induced in TAX1BP1 knocked down cells did increase cell death by 25–43% as compared to the siCon cells within each treatment group. As for NDP52 knockdown cells, MTAs resulted in cell death, but the percentages were comparable to control siRNA treated cells within each treatment group. This data suggests that mitochondrial dysfunction exacerbates cell death induced by the loss of TAX1BP1 in MDA-MB-231 cells.

### MitoQ treatment lead to the loss of NDP52 in SST2/SHR rat tumors that contain p53-positive protein aggregates

**Fig. 6:**
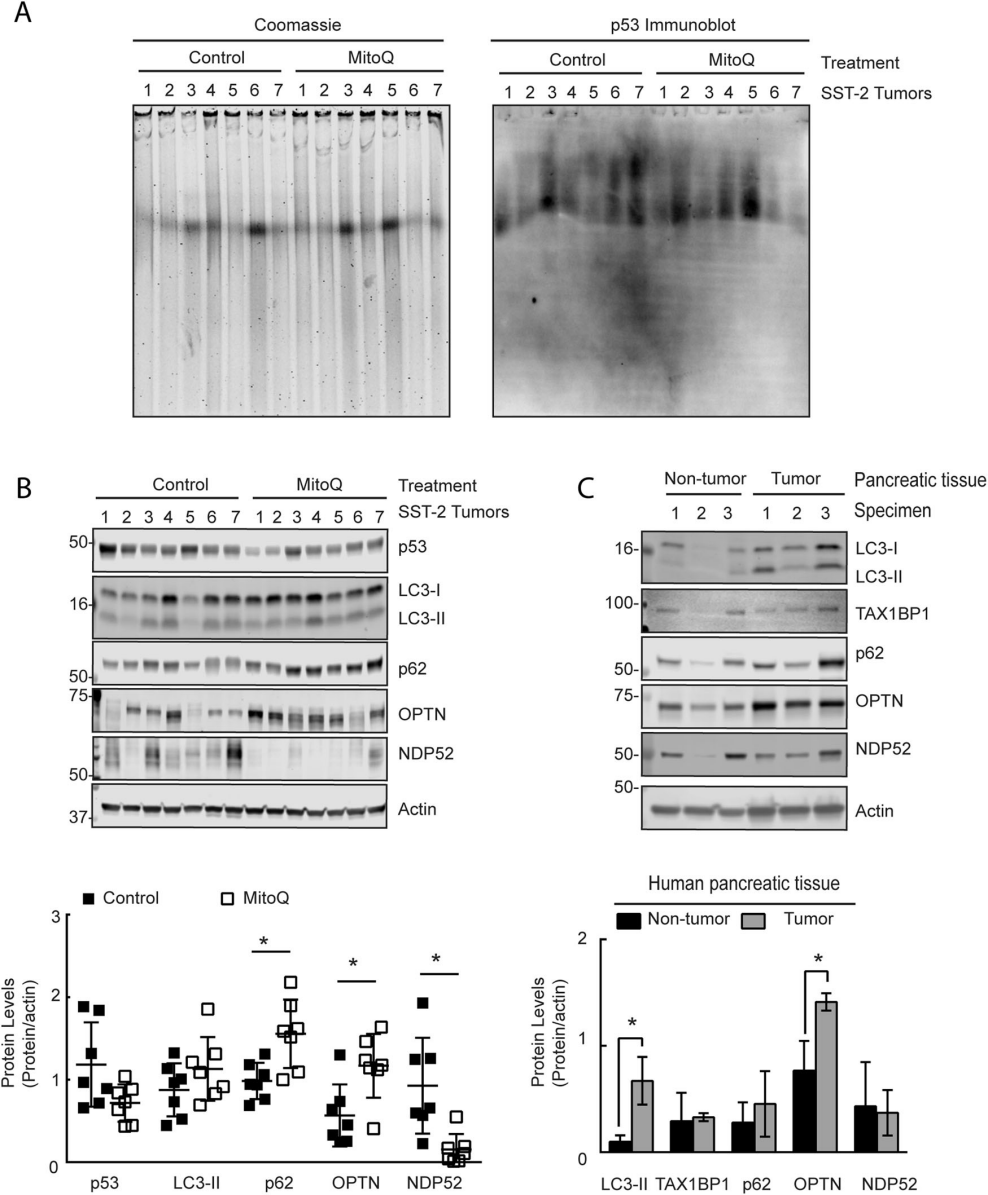
SHR/SST2 rat tumor tissue show p53 positive protein aggregates and loss of NDP52 with MitoQ. **a** Coomassie blue stained and p53 immunoblotted CNGs using total tissue lysates from rat breast tumor tissue extracted from SST-2 tumor-bearing spontaneous hypertensive rats at 14 days post saline (*n* = 7) or MitoQ (12.5 mg/kg) (*n* = 7) injections. **b** LC3 (*p* = 0.207), p53 (*p* = 0.047), p62 (*p* = 0.007), OPTN (*p* = 0.012), and NDP52 (*p* = 0.005) immunoblots from rat tumor tissue exposed to control (*n* = 7) or MitoQ (*n* = 7). (Student’s Test). **c** LC3 (*p* = 0.012), TAX1BP1 (*p* = 0.824), p62 (*p* = 0.449), OPTN (*p* = 0.018), and NDP52 (*p* = 0.828) immunoblots from human pancreatic non-tumor (*n* = 3) and tumor (*n* = 3) tissue (Student’s Test).

To extrapolate the findings from the cell lines into an in vivo model, the presence of p53 positive protein aggregates and autophagy receptors levels were investigated using a rat tumor tissue from a spontaneous hypertensive rat (SHR) that harbored SST-2 breast tumors^34,36^. Similar to human breast cancer biopsies^36,38^, rat breast tumors demonstrated p53 positive protein aggregates based on p53 immunoblotted CNG gels (Fig. 6a). Moreover, the protein levels of p53 had decreased in MitoQ-exposed tumor tissue as compared to the non-treated tumor tissue (Fig. 6b). As for the autophagy receptors, MitoQ-treated tumors had an increase in p62 and OPTN levels, and a decrease in NDP52 levels as compared to the control tumor tissue (Fig. 6b). Based on the in vitro findings (Fig. 3), NDP52 was dependent on the lysosome for degradation, which may suggest that autophagy could have caused the depletion of NDP52. These data demonstrate that NDP52 may be a potential endpoint to assess autophagy in vivo for both preclinical and clinical studies.

To further confirm the feasibility to assess autophagy receptors in human cancer tissue, protein lysates from human pancreatic non-tumor and tumor tissue were immunoblotted for LC3-II and autophagy receptor proteins (Fig. 6c). LC3-II did increase in the pancreatic tumor samples suggesting that pancreatic tumors have increased autophagy as compared to non-tumor tissue. Moreover, these results demonstrate that the autophagy receptor OPTN increased, while NDP52, TAX1BP1, and p62 remained comparable to the control. Using human cancer specimens, our data confirmed that these receptor proteins may assist in the characterization and monitoring of selective autophagy in patients that receive lysosomal neutralizing drugs.

## Discussion

Our study reveals mitochondrial dysfunction in cancer cells can cause the formation of p53 positive protein aggregates that accumulate due to insufficient aggregated-protein degradation. NDP52 was identified as an autophagic receptor that facilitates aggregate recognition and transport to impaired lysosomes in a manner that was dependent on TAX1BP1, and it was selectively being degraded until prolonged mitochondrial dysfunction caused a loss in TAX1BP1 levels. Depletion of TAX1BP1 suppressed NDP52 dependent aggrephagy and autophagic flux leading to cell death (Supplementary Fig. 6). The mechanisms contributing to MTA-induce aggregate formation and lysosomal impairment remain to be fully deciphered. MTAs induce the generation of free radicals that are a known cause the formation of aggregated proteins^18,39–41^, which warrants further investigation. As for lysosomal impairment, mitochondrial dysfunction has been proposed to impair lysosomal structure and activity^30^, but the mechanism(s) facilitating this relationship remain unknown. Here, we demonstrate that MTA-induced mitochondrial dysfunction is a relevant cancer model to further study this mitochondrial/lysosomal relationship.

Methods to evaluate autophagy modulation in clinical studies include quantifying autophagosome numbers, lysosomal enzymes, p62, and LC3^5,12,13,42–45^. Our findings indicate these methods may not identity dysfunctional aggrephagy or the accumulate of protein aggregates. To further investigate this response, the immunocompetent SST-2/SHR breast cancer tumor model was used to show p53 positive protein aggregates and that MTA treatment altered the protein levels of NDP52, OPTN and p62 in vivo. Similar to this preclinical model, human pancreatic tumor biopsies demonstrated changes in the autophagy receptor OPTN as compared to non-cancerous tissue, which can be leveraged for further investigation. Collectively, this study suggests that the levels of protein aggregates, TAX1BP1, NDP52, and OPTN are potential biomarkers to assess autophagy in preclinical and clinical studies that administer autophagy modulators and lysosomal inhibitors.

Bortezomib is previously reported to provide no clear benefit in metastatic breast cancer patients^2^. The clearance of misfolded proteins by autophagy is proposed to be a contributing factor leading to the lack of therapeutic efficacy^3,4,13,21^. Our findings indicate that cancer cells, in an environment that favors mitochondrial stress, generate p53 positive protein aggregates that resist lysosomal degradation. Several somatic mutations in *TP53* frequently occur in human tumors, including breast, that can form p53 protein aggregates to promote drug resistance^34,36,38^. We report that mitochondrial dysfunction, a known stress that leads to cytosolic acidosis^35^, can drive the “hot spot” TP53 missense mutated (R280K) protein to aggregate in MDA-MB-231 cells^34^. This mechanistic insight has the potential to be developed into a biologically-relevant biomarker to identify dysfunctional mitochondria and aggrephagy in patients that harbor mutations in the TP53 gene for personalized treatment options.

Autophagy contributes to several human diseases and the modulation of autophagy is a potential therapeutic strategy^5,46^. As new autophagy modulating agents emerge, robust and mechanistically-sound methods to evaluate these agents are required to assess autophagy modulation in the clinic^6^. In this study, multiple experimental models and primary tumor samples demonstrated that aggregated protein, TAX1BP1, and NDP52 may be sensitive markers for assessing lysosomal degradation of autophagic cargo for preclinical studies and clinical trials utilizing lysosomal neutralizing agents. In addition, this report demonstrates that differences in spatial measurements between autophagic proteins and cargo may have potential to evaluate autophagy using immunohistostaining. Collectively, this study demonstrated that mitochondrial dysfunction-induced, lysosomal-resistant protein aggregates and presents promising methods to further evaluate selective autophagy for preclinical and clinical studies.

## Methods and materials

### Tissue and cells

Human pancreatic and rat tumor tissues were homogenized to collect protein lysates. Female spontaneous hypersensitive rats (SHRs) were implanted with SST-2 implantation as previously described^47^. The human tissue study adhered to IRB-approved protocols at the University of Florida and the United States Food and Drug Administration, while the rat study was approved by IACUC at the FDA. All cell lines were obtained from ATCC and cultivated using their conditions. All cells were verified as mycoplasma free and cultured up to 10 passages. The mt-GFP plasmid was a kind gift from Pantelis Tsoulfas (Addgene #44385). Stable MDA-MB-231 cells expressing mt-GFP were generated using the Lenti-X HTX system following the manufacturer’s protocol (Clonetech, Mountain View, CA).

### Aggregation propensity factor measurement

The aggregation propensity factor was determined using the PROTEOSTAT® Aggresome detection kit (Enzo, Farmingdale, NY) as manufacture describes.

### Flow cytometry

Flow cytometry was performed using a BD LSRII (BD Biosciences, San Jose, CA). All analyses were performed using FlowJo software (Ashland, OR). Mt-mKeima was analyzed as previously described^18^. A full description of the flow methodology can be found in the Supplementary Materials and Methods.

### Immunostaining

Sequentially, cells were fixed, permeabilized, blocked with 5% bovine serum albumin (BSA), and incubated with primary antibodies overnight at a 1:100–500 ratio of antibody. Following over-night incubation, cells were incubated with the appropriate Alexa-Fluoro antibody (Thermofisher) for 1 h at 4 °C. All antibodies used for immunostaining can be found in the Supplementary Materials and Methods.

### Confocal and electron microscopy

Cell preparation and imaging for electron microscopy was performed as previously described^19^. Confocal microscopy was performed using an inverted Zeiss LSM 700 confocal microscope with an integrated humidified incubation chamber containing 5% CO2 at 37 °C for live cell imaging as previous described^18^. Detailed parameters, formulas, and calculations are listed in Supplementary Materials and Methods, and Supplementary Tables 1–7 as directed by the autophagy guideline manuscripts^7,28,29^.

### Micro-flow imaging

Lysed-cells were centrifuged at 14,000×*g* for 5 min at 4 °C. Supernatants were collected and quantified using a bicinchoninic acid (BCA) assay (Thermofisher). A 1 mg/mL protein concentration was established by diluting the supernatant. A 1.5 mL protein solution was used to perform micro-flow imaging on MFI 5000 imaging system (Protein Simple, San Jose, CA).

### Agarose-acrylamide composite native gel electrophoresis

Agarose-acrylamide composite native gel electrophoresis was performed with modifications as described^26^. Proteins (5 μg) were loaded onto the gel with HiMark, a high molecular weight protein standard (Thermofisher). Electrophoresis was performed at 4 °C in an ice bucker for 5–6 h based the loss of the 450 kDa protein marker from the gel. The gels were gently removed and stained with Coomassie Brilliant Blue G-250 as described by the manufacture.

### Immunoblotting, cellular fractionations, and immunoprecipitation

Immunoblotting was performed using the following primary antibodies at a 1:500 to 1:1000 ratio. Immunoblot images were collected using an Odyssey imager (LI-COR, Lincoln, NE) and densitometry was analyzed using Image J software (NIH, Bethesda, MD). Antibody information can be found in the Supplementary Materials and Methods. Fractionations and immunoprecipitation were performed as previous described^18,48^. Immunoprecipitation was performed using 2 μg of LC3-II antibody (Sigma) or mouse IgG (Cell Signaling, 5415). For soluble and insoluble fractionations, cells were trypsinized, washed in PBS, and incubated with 0.5% Triton-X for 20 min on ice followed by centrifugation at 4 °C for 10 min at 12,000×*g*. Supernatant was collect and used as the soluble fraction. The pellet was and lysed in to collect the supernatant, which was the insoluble fraction.

### Knockdown of TAX1BP and NDP52

Cells were seeded for 24 h prior to transfection using Dharmafect I as described by the manufacture (Dharmacon, T-2001, Lafayette, CO). ON-TARGET plus SMARTPOOL siRNA at 2 nM was used for the control, TAX1BP1 and NDP52 (Dharmacon, D-001810-10, LU-016892-00, and LU-010637-00). Cells were incubated for 24 h prior to the addition of any treatment. A dose-response was performed to identify the optimal concentrations of siRNA (Data not shown).

### Reagents and statistics

MitoQ and MitoApo were kind gifts from Drs. Joseph and Kalyanaraman at the University of Wisconsin Medical College. All other reagents and chemical were purchased from Sigma Aldrich. Statistical analyses were performed using GraphPad Prism 6 from at least three independently performed experiments and the coefficient of variance was determined. A student’s *T* test was performed for differences in a single parameter between two conditions. When >2 treatments were used, a one-way ANOVA was performed with a Tukey’s or Dunnett’s multiple comparison test to identify statistical differences. When >2 groups were used, a two-way ANOVA was performed with a Tukey’s multiple comparison post-hoc test to identify significant differences. No variation was identified within each group. A *p* value of <0.05 indicated statistical differences. All the main results of the statistical analyses are listed in Supplementary Tables 7, 8, 9 and 10.

## Supplementary information

Supplementary Figure Legends

Supplementary Figure 1

Supplementary Figure 2

Supplementary Figure 3

Supplementary Figure 4

Supplementary Figure 5

Supplementary Fgure 6

Supplementary Information

Supplemental Table 1

Supplemental Table 2

Supplemental Table 3

Supplemental Table 4

Supplemental Table 5

Supplemental Table 6

Supplemental Table 7

Supplemental Table 8

Supplemental Table 9

Supplemental Table 10
