# Supplementary material for: Mitochondrial dysfunction generates aggregates that resist lysosomal degradation in human breast cancer cells
Source: Cell Death Dis. 2020 Jun 15;11(6):460. doi: 10.1038/s41419-020-2658-y (PMC7296005; doi:10.1038/s41419-020-2658-y)
Supplement: Supplementary file 1 — Supplementary Figure Legends [file 41419_2020_2658_MOESM1_ESM.docx]

**Supplementary Table 10:** Parameters, figure locations, degrees of freedom, and p values for Student’s T tests.

| Student T Tests | | | |
| --- | --- | --- | --- |
| Parameter | Figure location | DF | p Value |
| PINK1 levels | Supplementary Figure 5A | 4 | 0.0019 |
| Cell Death | Supplementary  Figure 5B | 4 | 0.00015 |
| p53 levels | Figure 6B | 12 | 0.04729 |
| LC3-II levels | Figure 6B | 12 | 0.20765 |
| p62 levels | Figure 6B | 12 | 0.00740 |
| OPTN levels | Figure 6B | 12 | 0.01195 |
| NDP52 levels | Figure 6B | 12 | 0.00580 |
| LC3-II levels | Figure 6C | 4 | 0.01293 |
| TAX1BP1 levels | Figure 6C | 4 | 0.82457 |
| p62 levels | Figure 6C | 4 | 0.44908 |
| OPTN levels | Figure 6C | 4 | 0.01824 |
| NDP52 levels | Figure 6C | 4 | 0.82848 |
