# Supplementary figures and images for: Mitochondrial dysfunction generates aggregates that resist lysosomal degradation in human breast cancer cells
Source: Cell Death Dis. 2020 Jun 15;11(6):460. doi: 10.1038/s41419-020-2658-y (PMC7296005; doi:10.1038/s41419-020-2658-y)

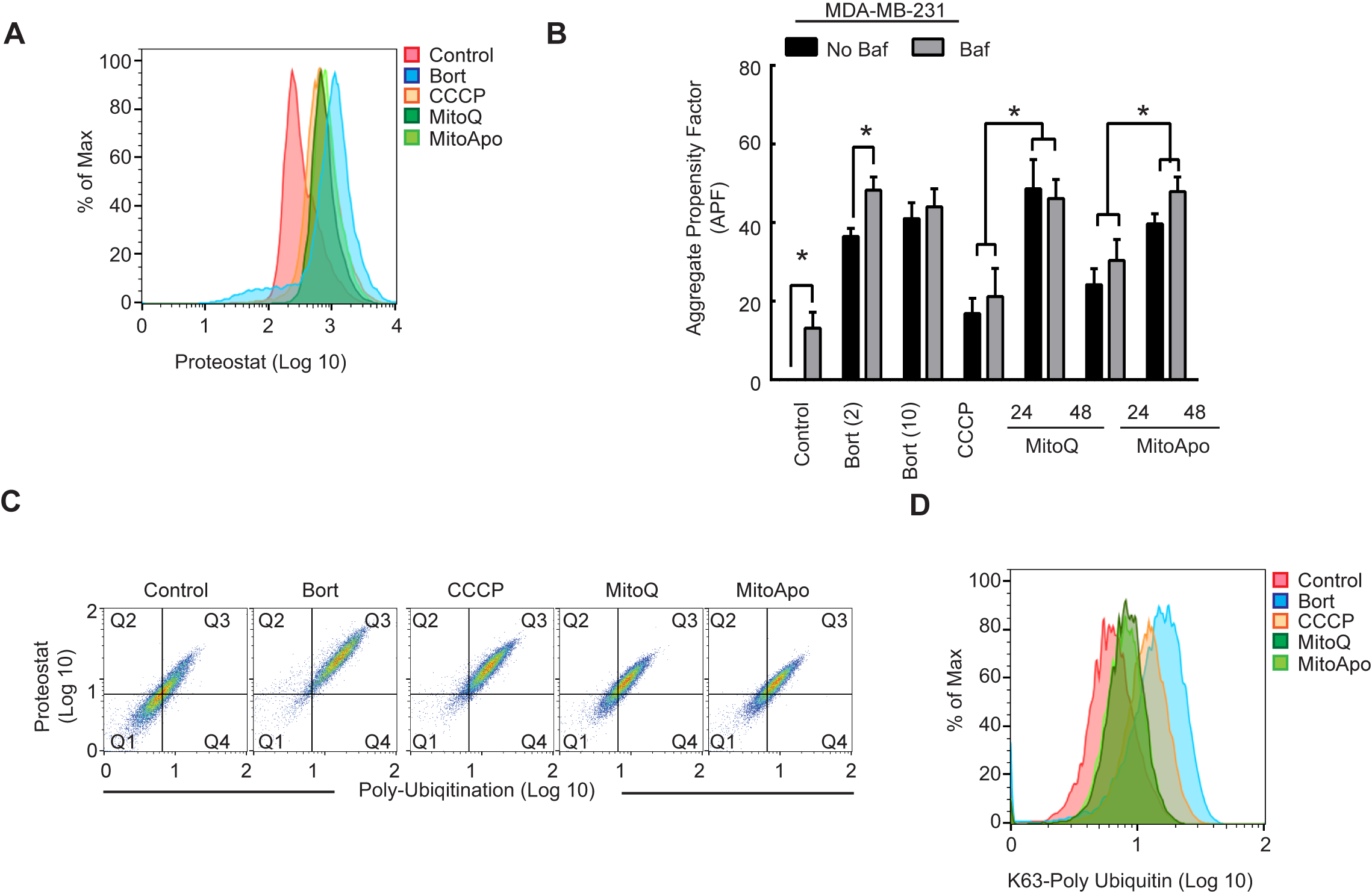

Supplement: Supplementary file 3 — Supplementary Figure 2 [file 41419_2020_2658_MOESM3_ESM.tif]

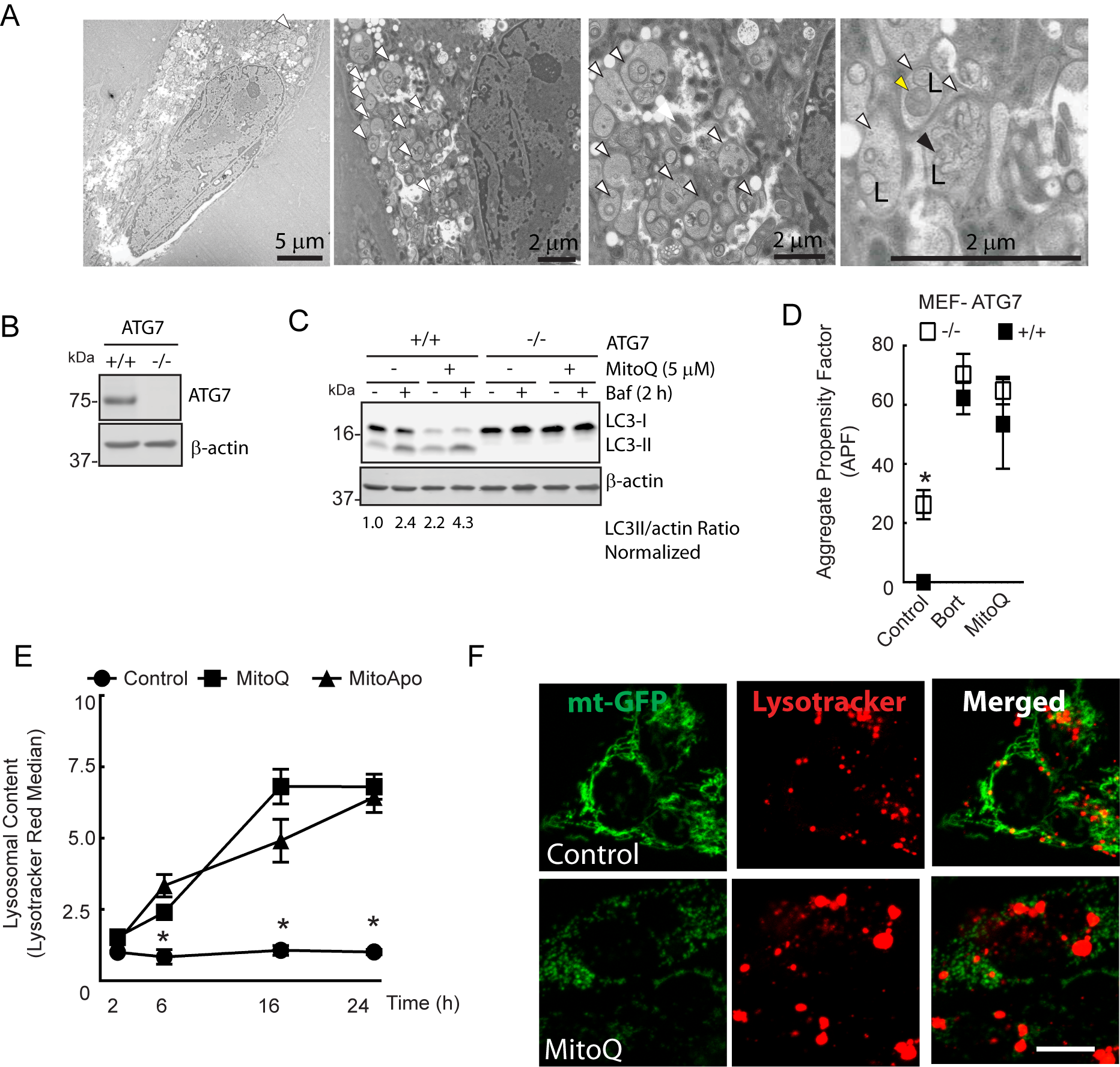

Supplement: Supplementary file 4 — Supplementary Figure 3 [file 41419_2020_2658_MOESM4_ESM.tif]

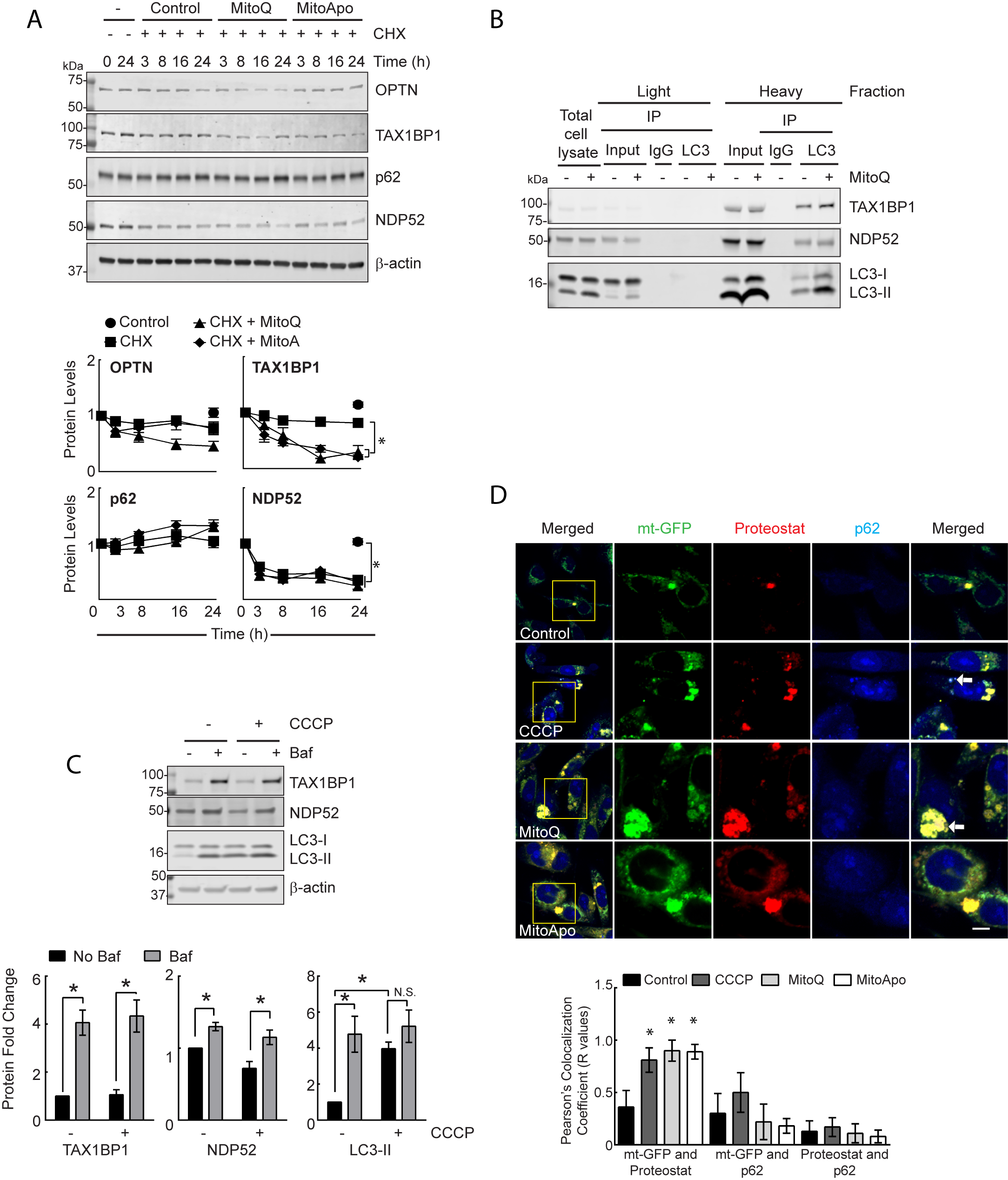

Supplement: Supplementary file 5 — Supplementary Figure 4 [file 41419_2020_2658_MOESM5_ESM.tif]

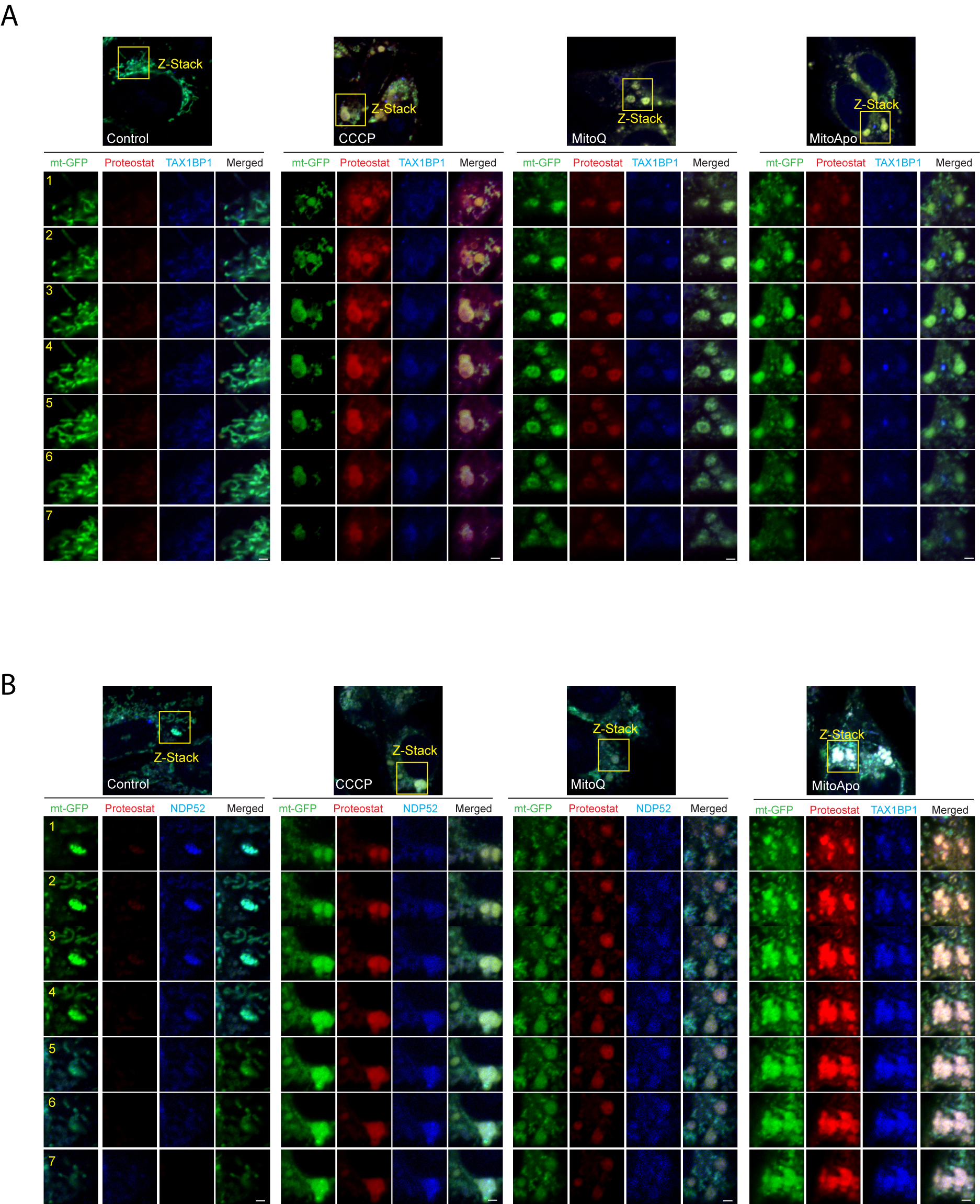

Supplement: Supplementary file 6 — Supplementary Figure 5 [file 41419_2020_2658_MOESM6_ESM.tif]

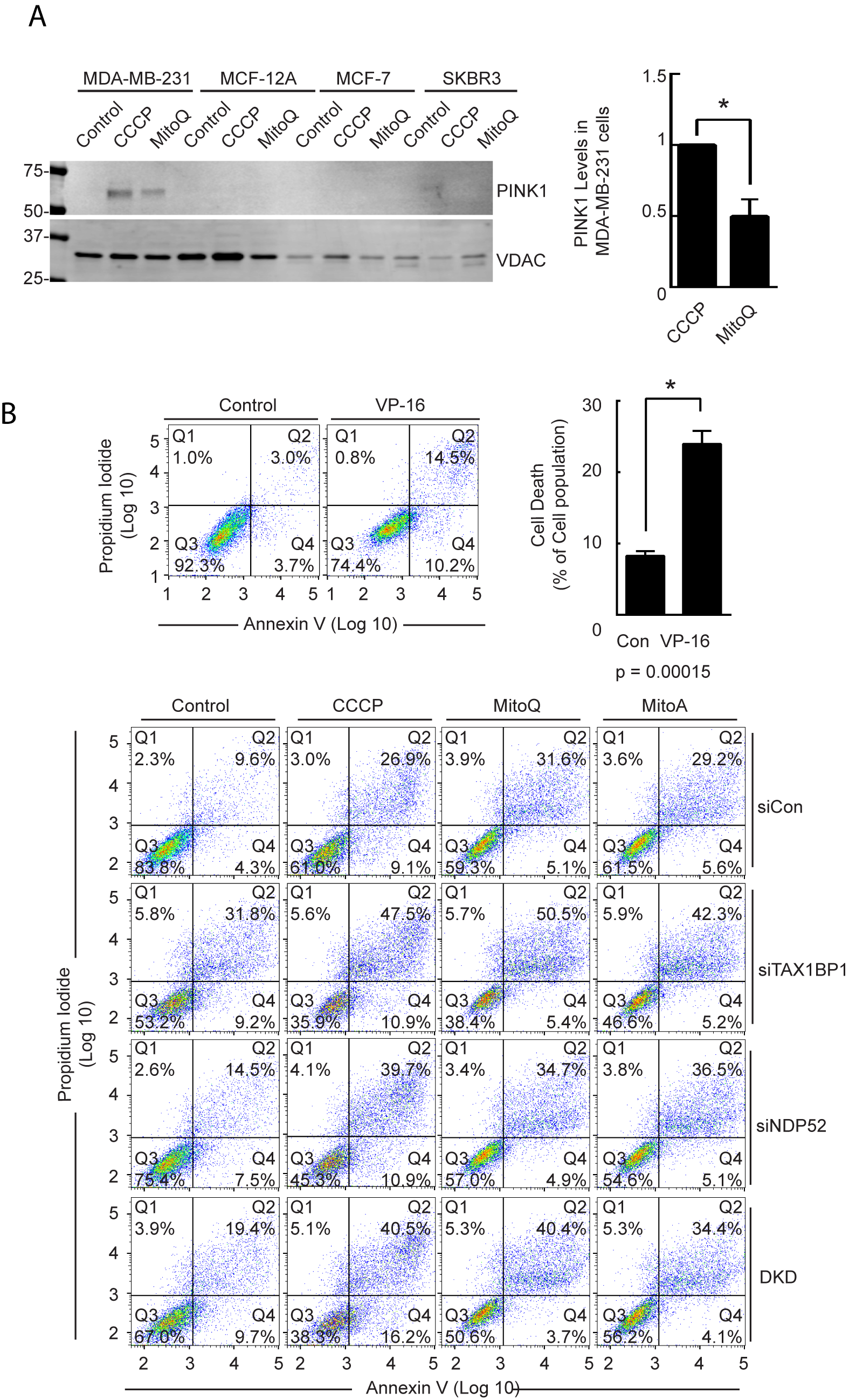

Supplement: Supplementary file 7 — Supplementary Fgure 6 [file 41419_2020_2658_MOESM7_ESM.tif]

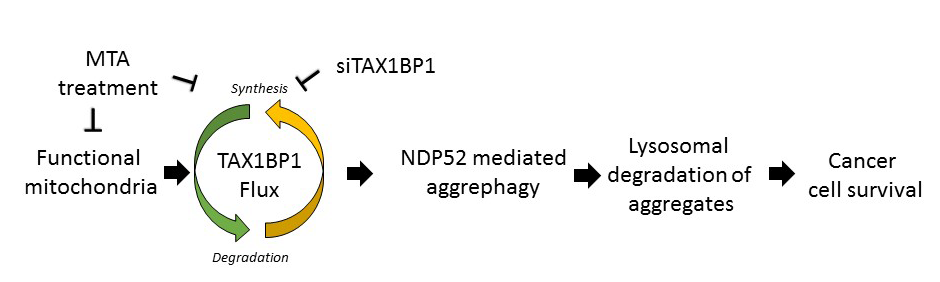

Supplement: Supplementary file 8 — Supplementary Information [file 41419_2020_2658_MOESM8_ESM.tif]
