# Supplementary material for: Mitochondrial dysfunction generates aggregates that resist lysosomal degradation in human breast cancer cells
Source: Cell Death Dis. 2020 Jun 15;11(6):460. doi: 10.1038/s41419-020-2658-y (PMC7296005; doi:10.1038/s41419-020-2658-y)
Supplement: Supplementary file 9 — Supplemental Table 1 [file 41419_2020_2658_MOESM9_ESM.docx]

**Supplementary Information**

**Supplementary Materials and Methods.**

**Flow Cytometry**Cell death was assessed using the FITC Annexin V FITC apoptosis detection Kit I as described by manufacture (BD Biosciences, 556547, San Jose, CA). Lysotracker Red (200 nM) (Thermo Fisher, L7528) was incubated for 20 minutes in trypsinized cells at 37^o^C followed by two PBS washes prior to FACs analysis using the 560 nm excitation laser and 610/20 emission filter. Live mt-GFP cells were trypsinized, washed twice in PBS, analyzed using the 488 nm excitation laser and a 510/20 emission filter, while fixed p53 immuno-stained cells were analyzed under similar conditions. For the aggregate propensity factor, Proteostat-stained cells were collected using a 488 nm excitation laser and a 610/20 nm emission filter. As previously described ^1^, the aggregation propensity factor (APF) was calculated using the equation below

APF = 100 × ((MFI_treated_ − MFI_control_)/MFI_treated_), wherein MFItreated and MFIcontrol are the mean fluorescence intensity values from control and treated samples.

For the cell population analysis of poly-ubiquitin and Proteostat, cells were immune-stained with a poly-ubiquitin (K63 linkage) antibody (Abcam, ab179434, Cambridge, MA) and Alexa-Fluor 488 secondary antibody (Thermofisher, Waltham, MA) prior to Proteostat staining and FACs analysis using the 488 nm excitation laser with 530/10 and 610/20 nm emission filters.

**Immunostaining**Primary antibodies that were used include: poly-ubiquitin (K63 linkage) antibody (Cell signaling, 5621S, Danvers, CO), LC3B (Sigma Aldrich, SAB-4200361, St. Louis, MO), p53 (Cell signaling, 2524, Danvers, MA), LAMP1 (Cell signaling, 9091), TAX1BP1(Cell Signaling, 5105), and NDP52 (Cell Signaling, 60732). Anti-Rabbit and Anti-Mouse Alexa-Fluoro antibodies were used with 405 nm and 488 nm excitations (Thermo Fisher, A32723, A31556, and A31553).

**Confocal Imaging**
IgG immune-stained controls were used to establish the detection parameters for immunofluorescence using the range indictor to set digital gain and offset, and Proteostat-stained CCCP- or Bort-treated cells were used to establish the upper baseline for the detection parameters and image capture. Single fields (5-10 total per treatment), and 5 x 5 tiles (3-8 different positions) were collected. Minimum and maximum levels of the individual channels were adjusted in a consistent uniformed manner between all the treatment groups to improve the picture quality for publication using ZEN Black or Blue (Zeiss, Oberkochen, Germany). Images analysis for spatial measurement using areal quantification was performed using the ZEN blue service I software package (Zeiss). Thresholds were established using CCCP-treated cells and applied to all images.

**Immunoblotting**
Primary antibodies used were : mouse and rabbit actin, tubulin, VDAC, LAMP1, GFP, p53, TAX1BP1, NDP52 (Cell Signaling, Cat # 4970, 58069, 4661, 15665, 29565, 25245, 5105, 607325), mt-RNA Polymerase, p62 (Abcam, Cat# 32988, 56416), Cathepsin D (Invitrogen, 17236, ATG7 (Sigma Aldrich, A2856), LC3B (Novus, NB100-2220, Littleton, CO), OPTN (Protein Tech, 10837-1-AP, Rosemont, IL) and LAL (Santa Cruz, 58374, Dallas, TX).

**Mitochondrial Extraction**
A crude mitochondrial extraction was performed to obtain for PINK1 immunoblotting as previously described^2^. Briefly, twenty to forty million cells were subjected to a hypotonic solution prior to homogenization. The homogenate underwent differential centrifugation to obtain a crude mitochondrial pellet that was lysed with radioimmunoprecipitation buffer.

**References:**

1 Shen, D. *et al.* Novel cell- and tissue-based assays for detecting misfolded and aggregated protein accumulation within aggresomes and inclusion bodies. *Cell Biochem Biophys* **60**, 173-185, doi:10.1007/s12013-010-9138-4 (2011).

2 Biel, T. G. & Rao, V. A. Mitochondrial dysfunction activates lysosomal-dependent mitophagy selectively in cancer cells. *Oncotarget* **9**, 995-1011, doi:10.18632/oncotarget.23171 (2018).

**Supplementary Figure Legends**

**Supplemental Figure 1: Mitochondrial dysfunction impairs lysosomal dependent aggregate degradation.** (**A**) Representative histogram of Proteostat in MDA-MB-231 cells treated with Bort (2 nM) and CCCP (30 μM) for 24 hours or MitoQ (1 μM) or MitoApo (1 μM) for 48 hours using FACs analysis. (**B**) MDA-MB-231 cells were exposed to DMSO (control), Bort(2) (2 nM), Bort(10) (10 nM), and CCCP (30 μM) for 24 hours or MitoQ (1 μM), and MitoApo (1 μM) for 24 or 48 hours with or without Baf (5 nM) for the final 2 hours to determine the aggregation propensity factor using FACS analysis. (**C**) Representative scatter plots of K-63 immune-labeled and Proteostat-stained MDA-MB-231 cells subjected to DMSO (Control), Bort, CCCP, MitoQ, or MitoApo treatment for 16 hours. Bars represent the mean and the error bars are SEM. (two-way ANOVA, *n=3-5*, *p < 0.05 according to Tukey’s post hoc test). (**D**) Representative histogram of K-63 poly-ubiquitin FACs analysis of K-63 poly-ubiquitin immuno-stained MDA-MB-231 cells treated with CCCP (30 μM), MitoQ (1 μM) or MitoApo (1 μM) for 16 hours.

**Supplemental Figure 2: Confirmation of aggregate-like structures in lysosomes, defective lysosomal degradation, and lysosomal analysis using lysotracker analyses.** (**A**) Electron micrograph of MitoQ-treated MDA-MB-231 cells for 24 hours. White, yellow, and black arrow heads identify single membrane bound irregularly shaped lysosomes (L), mitochondria remnants, and potential aggregate-fibril structures, respectively. (**B**) Immunoblot analysis of ATG7 from WT (+/+) and ATG7 knockout (-/-) MEF cells. (**C**) LC3 Immunoblot in WT and ATG7 knockout MEF cells treated with MitoQ (5 μM) in the presence and Bafilomycin (5 nM) for 24 hours. (**D**) Cells were exposed to Bort (5 nM) and MitoQ (5 μM) for 24 hours to quantify the APF (*n=4* per cell type). (**E**) Line graph of the mean fluorescent intensity of lysotracker red (200 nM) in MDA-MB-231 cells at the indicated time points following MitoQ (1 μM) and MitoApo (1 μM) treatments. (Repeated measure (Time) two-way ANOVA, n=3, *p<0.05 as indicated by a Turkey’s post comparison test.) (**F**) Live cell images of lysotracker red in mt-GFP expressing MDA-MB-231 cells with and without MitoQ (1 μM) treatment at 24 hours.

**Supplemental Figure 3: CCCP-treated MDA-MB-231 cells degrade NDP52 and TAX1BP1 via the lysosome in the presence LC3-II turnover inhibition.** (**A**) Representative immunoblots of the autophagic receptor proteins OPTN, TAX1BP1, p62, and NDP52 from MDA-MB-231 cells exposed to MitoQ (1 μM) or MitoApo (1 μM) in the presence or absence of cycloheximide (CHX) (25 μM) at the indicated times (ANOVA at 24 hours per protein, *n=3-5*, *p < 0.05 as according to Tukey’s post-hoc between treatments.) (**B**) Representative TAX1BP1 and NDP52 immunoblots of an autophagosome enhanced fraction (150 μg) after immunoprecipitation with LC3 from MDA-MB-231 cells treated with MitoQ for 24 hours. (**C**) Representative immunoblots of TAX1BP1, NDP52, and LC3 from MDA-MB-231 cells treated with and without CCCP (30 μM) in the presence and absence of Bafilomycin (5 nM) for 24 hours. (two-way ANOVA per protein, n=3, *p<0.05 as indicated by a Turkey’s post comparison test.) (**D**) Representative images of p62 immuno-labeled mt-GFP expressing MDA-MB-231 cells stained with Proteostat. Cells were treated with DMSO (Control), CCCP (30 μM), MitoQ (1 μM) or MitoApo (1 μM) for 24 hours. Scale bar is 5 μm. Whole cells were used to establish the Pearson’s correlation coefficient (R values shown) between mt-GFP, autophagic receptor, and Proteostat. (ANOVA per comparison, *n = 3-8 fields (5x5 tiles per field),* *p< 0.05 according to Tukey’s post-hoc test identified significant differences between the control and indicated treatment.) N.S. = Not significant. Bars represent mean and error bars are SEM.

**Supplemental Figure 4: NDP52 and TAX1-BP1 partly localize with aggregated mitochondria in MDA-MB-231 cells.** Z-sections of (**A**) TAX1BP1, and (**B**) NDP52 from mt-GFP expressing MDA-MB-231 cells stained with Proteostat following control (DMSO), CCCP (30 μM), MitoQ (1 μM), and MitoApo (1 μM) for 24 hours. Scale bar is 2 μm.

**Supplemental Figure 5: The lack of PINK1 accumulation and knockdown of TAX1BP1 and NDP52 does not affect the delivery of mitochondria to the lysosome.** (A) Immunoblot of PINK1 levels from 25 μg of crude mitochondrial extracts from different breast cell lines. (Student’s T Test, n=3, *p<0.05) (B)Representative FACs divided scatter plots of annexin V and propidium iodide fluorescence in MDA-MB-231 cells were treated with DMSO or VP-16 (10 μM) for 48 hours (Student’s T test, n=3, *p = 0.001), and TAX1BP1 and NDP52 knocked-down after CCCP (30 μM) treatment for 24 hours or MitoQ (1 μM) and MitoApo (1 μM) treatments for 72 hours Bars represents the mean and error bars are SEM.

**Supplemental Figure 6: Working model of aggrephagy in MDA-MB-231 cells with and without mitochondrial dysfunction.** MDA-MB-231 cells without MTA treatment are actively synthesizing and degrading TAX1BP1, which mediates NDP52 dependent aggrephagy with functional lysosomes to degrade aggregates and promote cell survival. Knocking down TAX1BP1 or treating the cells with MTAs caused a loss in TAX1BP1 levels that blocked NDP52 mediated aggrephagy leading to cell death. The loss of NDP52 mediated autophagy was not found to be the primary cause of protein aggregate accumulation in MTA-treated cells. In contrast to NDP52, LC3-II and TAX1BP1, these protein aggregates were not undergoing lysosomal degradation due to impaired activity that requires further investigation.

**Supplemental Figure 1**

**
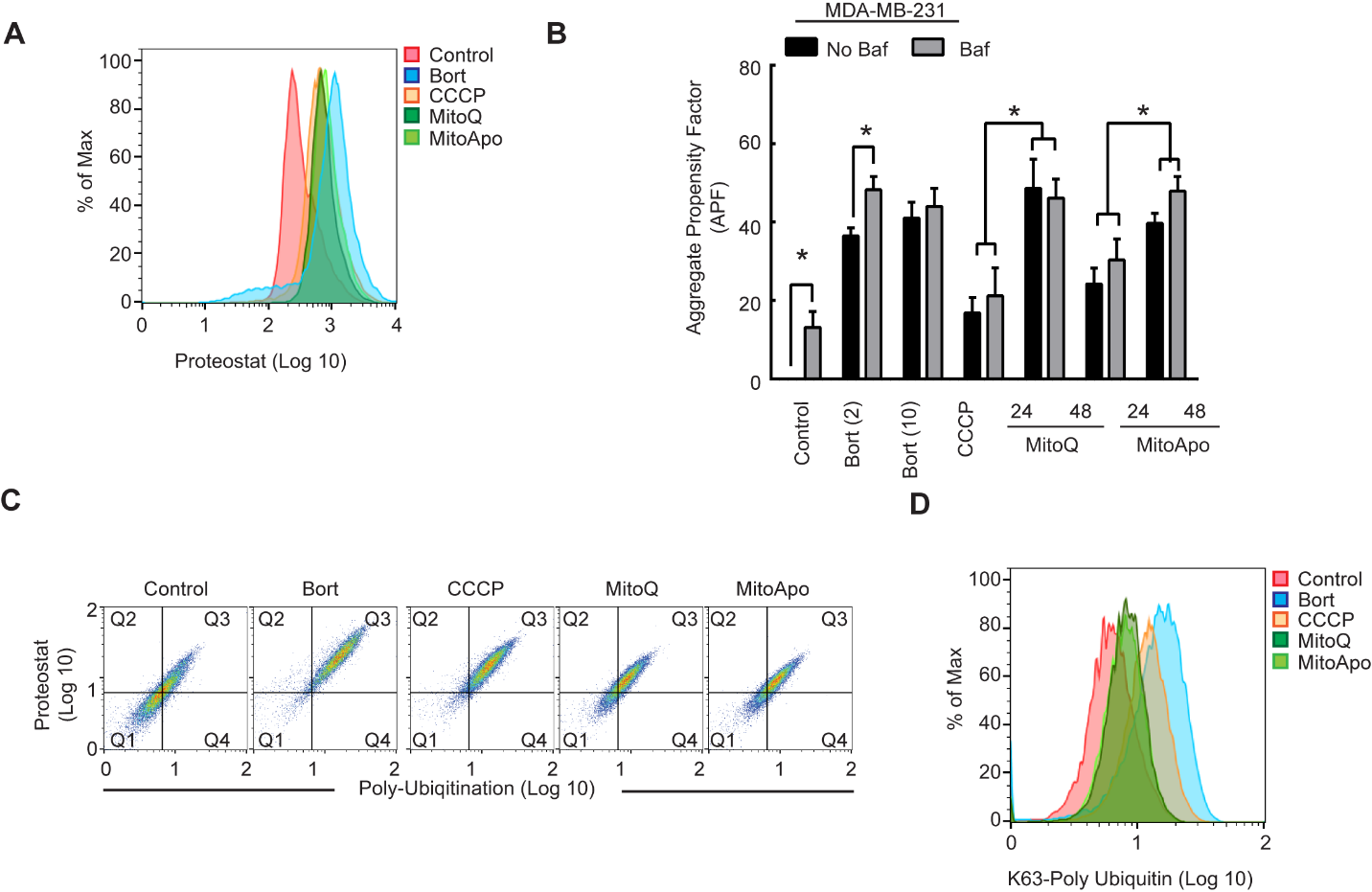
**

**Supplemental Figure 2**

**
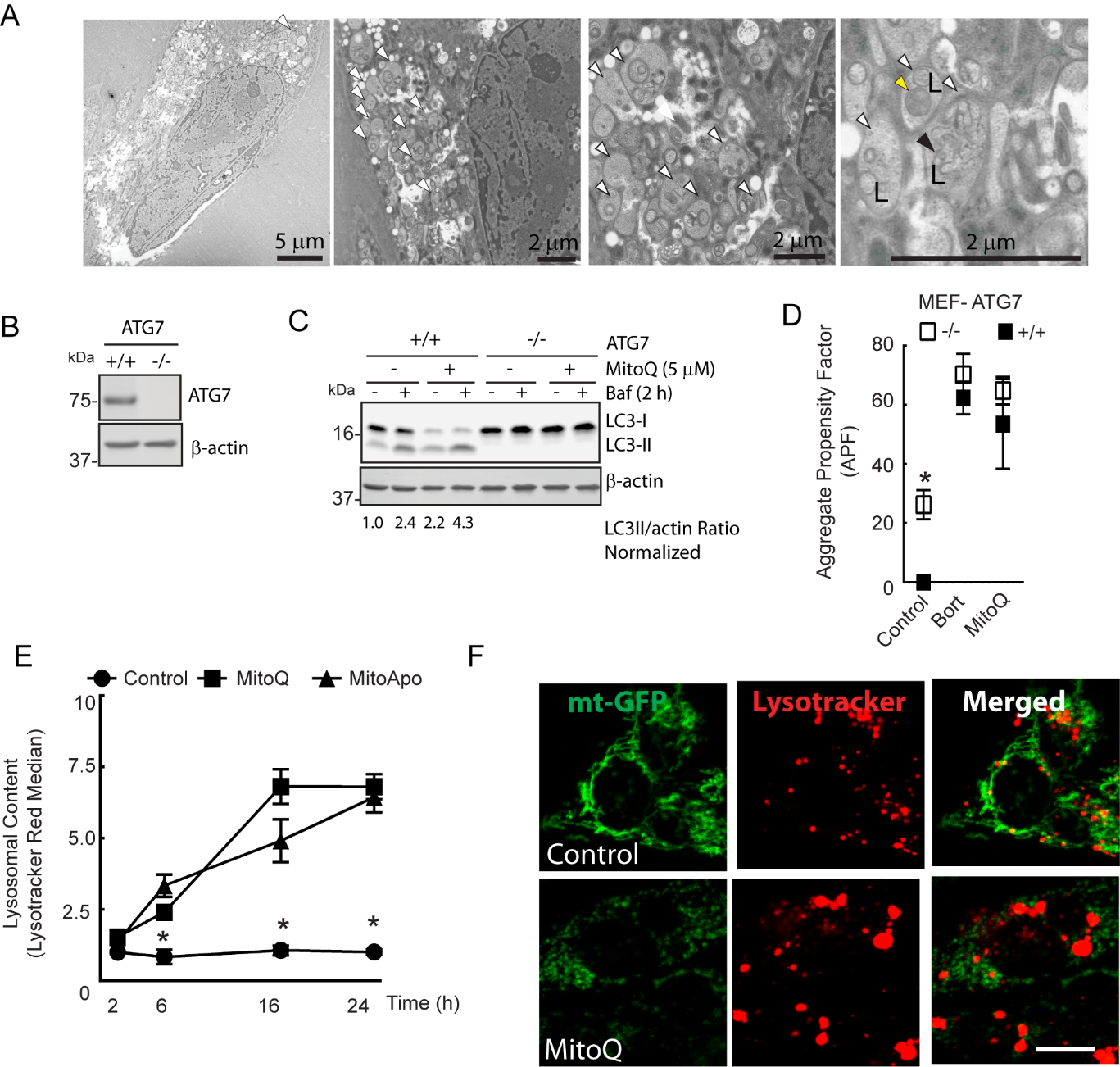
**

**Supplemental Figure 3**

**
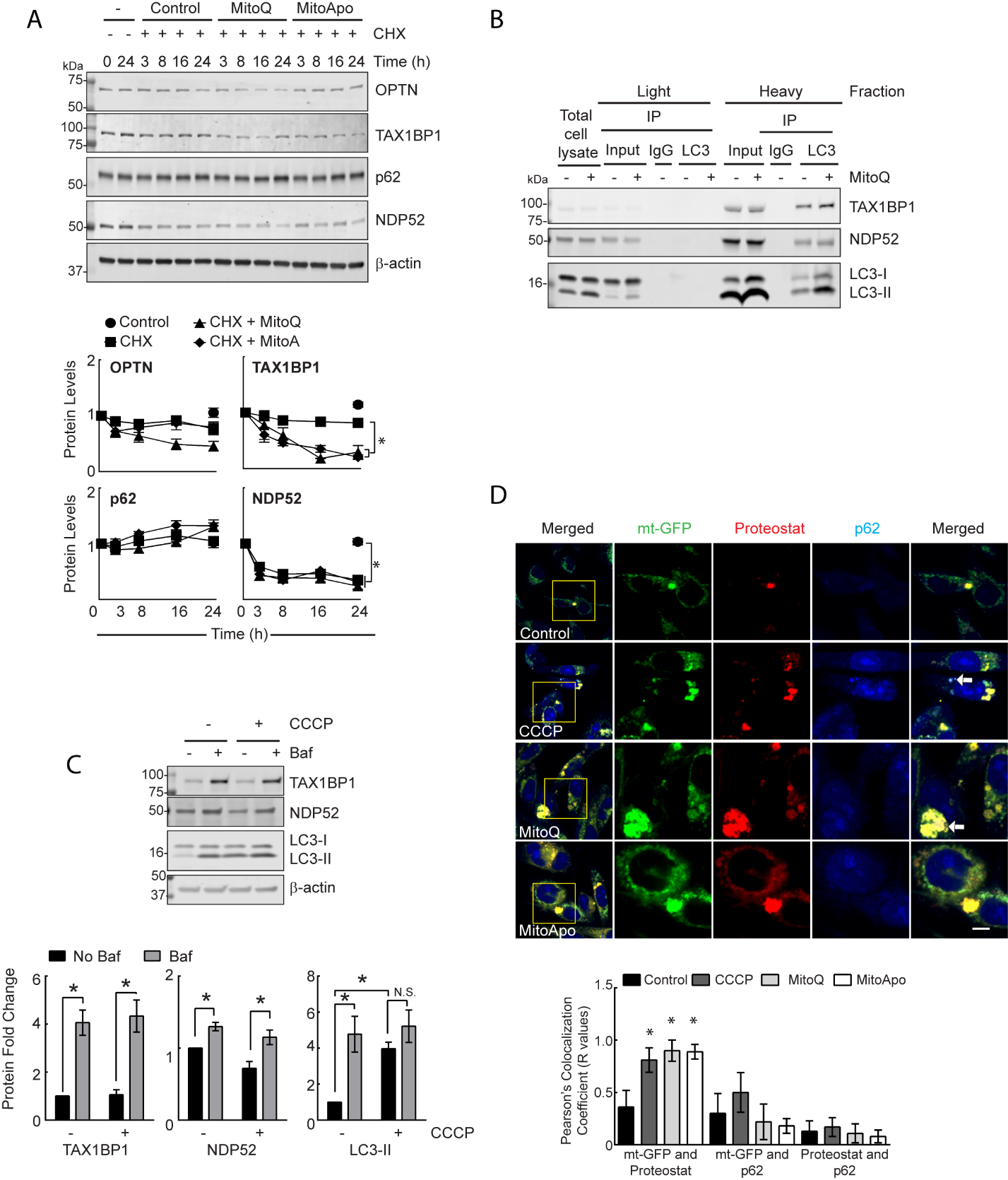
**

**Supplemental Figure 4**

**
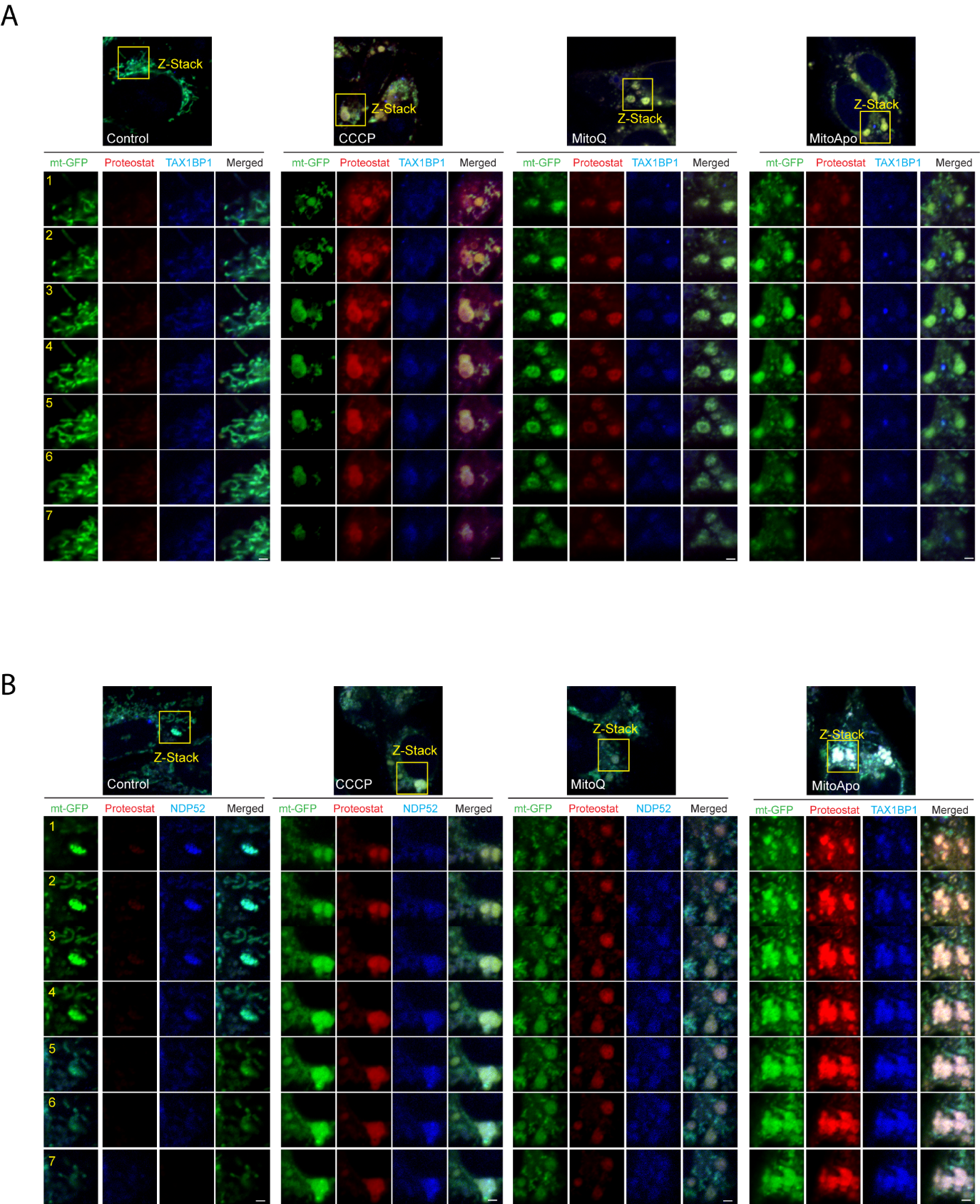
**

**Supplemental Figure 5**

**
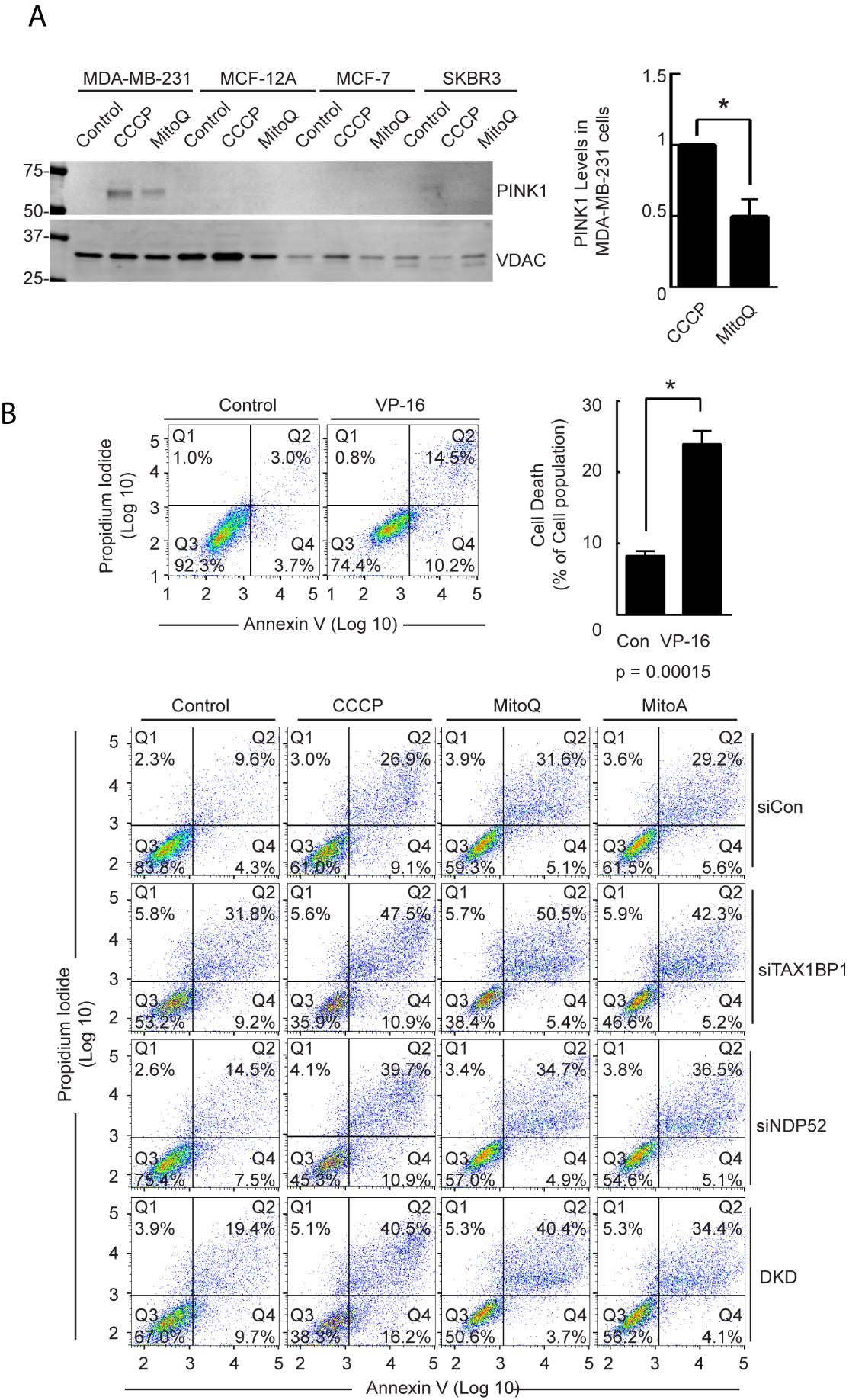
**

**Supplemental Figure 6**

**
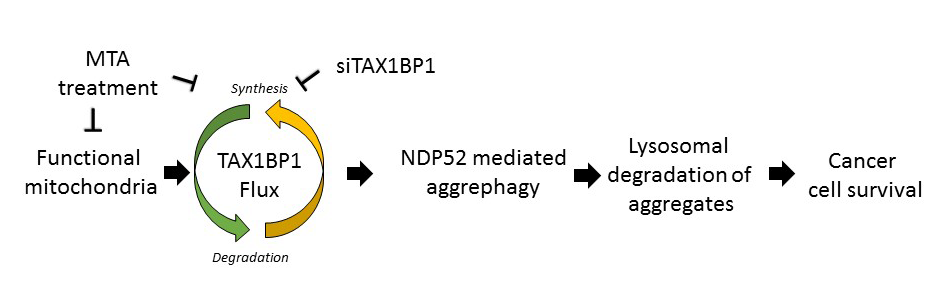
**

**Supplementary Table 1:** LC3 and Proteostat punctae and area quantification in MDA-MB-231 cells based on the number of punctae per cell.

| **Punctae analyses of MDA-MB-231 cells stained for LC3 and aggregates** | | | | | | | | | | | | | |
| --- | --- | --- | --- | --- | --- | --- | --- | --- | --- | --- | --- | --- | --- |
|  | ***Cell Count*** | ***Pearson's Correlation (R values)*** | | ***Population %  positive for  colocalized punctae*** | | ***GFP-LC3 punctae  per cell*** | | ***Proteostat punctae  per cell*** | | ***% of GFP-LC3  punctae with Proteostat*** | | ***% of Proteostat punctae  without GFP-LC3*** | |
| ***Treatment*** |  | Ave. | S.D. | Ave. | S.D. | Ave. | S.D. | Ave. | S.D. | Ave. | S.D. | Ave. | S.D. |
| ***Control*** | 529 | 0.27 | 0.115 | 23 | 6.1 | 6.49 | 1.93 | 2.91 | 0.9 | 36.02 | 1.25 | 19.15 | 1.56 |
| ***CCCP*** | 481 | 0.56* | 0.062 | 53* | 7.3 | 5.33 | 1.82 | 3.71 | 0.37 | 63.15* | 4.4 | 10.83 | 9.88 |
| ***MitoQ*** | 461 | 0.74* | 0.023 | 67* | 5.8 | 13.85* | 2.29 | 9.34* | 1.37 | 60.85* | 5.45 | 9.74 | 8.12 |
| ***MitoApo*** | 583 | 0.65* | 0.065 | 62* | 6.6 | 9.83* | 0.87 | 6.79* | 1.36 | 64.71* | 8.7 | 5.9 | 0.65 |
| ***Formula in Supplemental. Table 7*** | | - | | 8a | | 8b | | 8c | | 8d | | 8e | |
| **Area analyses of MDA-MB-231 cells stained for LC3 and aggregates** | | | | | | | | | | | |  |  |
|  |  | ***Area per  cell(AC) (μm^2)*** | | ***% of area with GFP-LC3 punctae*** | | ***% of area with Proteostat punctae*** | | ***LC3 punctae (μm^2)*** | | | |  |  |
|  |  |  |  |  |  |  |  | ***without  Proteostat (n=350)*** | | ***with  Proteostat  (n = 350)*** | |  |  |
| ***Treatment*** | | Ave. | S.D. | Ave. | S.D. | Ave. | S.D. | Ave. | S.D. | Ave. | S.D. |  |  |
| ***Control*** | | 825.42 | 29.61 | 2.23 | 0.82 | 0.8 | 0.27 | 1.7 | 1.7 | 5.29# | 7.11 |  |  |
| ***CCCP*** | | 458.89* | 122.18 | 7.97* | 1.06 | 4.69* | 0.58 | 1.19 | 1.07 | 15.44#* | 11.71 |  |  |
| ***MitoQ*** | | 827.73 | 163.52 | 9.91* | 1.49 | 4.71* | 1.05 | 1.25 | 1.07 | 22.22#* | 12.38 |  |  |
| ***MitoApo*** | | 749.8 | 85.64 | 10.65* | 4.02 | 5.79* | 2.86 | 1.27 | 1.06 | 28.66#* | 18.91 |  |  |
| ***Formula in Supplemental Table 7*** | | 8f | | 8g | | 8h | | 8i | | 8j | |  |  |

One- and two-way ANOVA, n=4-7 fields per group, *p<0.05 as indicated by a Tukey’s comparison test to the control, while #p<0.05 between groups. Ave = Average, and S.D. = Standard Deviation.

**Supplementary Table 2:** LAMP1 and Proteostat quantification in MDA-MB-231 cells based on area per cell.

| **Area analyses of MDA-MB-231 cells stained for LAMP1A and aggregates** | | | | | | | | | | | | | |
| --- | --- | --- | --- | --- | --- | --- | --- | --- | --- | --- | --- | --- | --- |
|  | ***Cell Count*** | ***Area  per cell (mm^2)*** | | ***Pearson's Correlation  (R values)*** | | ***Population %  positive for  colocalized punctae*** | | ***% of area with LAMP1*** | | ***% of area with Proteostat punctae*** | | ***% of Proteostat punctae within  LAMP1*** | |
|  |  |  |  |  |  |  |  |  |  |  |  |  |  |
| ***Treatment*** |  | Ave. | S.D. | Ave. | S.D. | Ave. | S.D. | Ave. | S.D. | Ave. | S.D. | Ave. | S.D. |
| ***Control*** | 415 | 873.17 | 29.21 | 0.17 | 0.07 | 23.59 | 11.53 | 12.74 | 3.14 | 1.36 | 0.56 | 96.43 | 3.23 |
| ***CCCP*** | 252 | 698.91* | 175.63 | 0.68* | 0.12 | 90.41* | 4.5 | 19.12* | 1.57 | 6.43* | 0.89 | 98.98 | 0.68 |
| ***MitoQ*** | 368 | 855.91 | 75.56 | 0.73* | 0.09 | 47.85* | 8.09 | 21.03* | 5.32 | 5.19* | 2.24 | 98.84 | 0.61 |
| ***MitoApo*** | 455 | 861.09 | 70.45 | 0.68* | 0.12 | 58.36* | 11.29 | 23.99* | 6.69 | 10.23* | \| 4.05 \| \| --- \| | 99.69 | 0.25 |
| ***Formulas in Supplemental Table 7*** | | 8f | | - | | 8k | | 8l | | 8h | | 8m | |

One-way ANOVA, n=4-8 fields per group, *p<0.05 as indicated by a Tukey’s comparison test to the control, while #p<0.05 between groups. Ave = Average, and S.D. = Standard Deviation.

**Supplementary Table 3:** Proteostat and p53 quantification of MDA-MB-231 cells based on area per cell.

| **Punctae and area analyses of MDA-MB-231 cells stained for p53 and aggregates** | | | | | | | | | | | | | |
| --- | --- | --- | --- | --- | --- | --- | --- | --- | --- | --- | --- | --- | --- |
|  | ***Cell Count*** | ***% of cells positive for colocalization*** | | ***Pearson Correlation  (R values)*** | | ***Colocalized punctae per cell*** | | ***Area of  p53 per cell  (μm^2)*** | | ***Area of  Proteostat punctae per cell (μm^2)*** | | ***% of p53 area with  Proteostat*** | |
|  |  |  |  |  |  |  |  |  |  |  |  |  |  |
| ***Treatment*** |  | Ave. | S.D. | Ave. | S.D. | Ave. | S.D. | Ave. | S.D. | Ave. | S.D. | Ave. | S.D. |
| ***Control*** | 437 | 0.12 | 0.06 | 0.13 | 0.04 | 0.08 | 0.08 | 594.37 | 57.58 | 1.77 | 1.65 | 0.291 | 0.267 |
| ***CCCP*** | 588 | 0.83* | 0.053 | 0.67* | 0.06 | 4.87* | 0.98 | 695.36 | 76.88 | 65.21* | 10.88 | 9.41* | 1.42 |
| ***MitoQ*** | 383 | 0.59* | 0.07 | 0.69* | 0.08 | 2.64* | 0.49 | 677.46 | 49.65 | 30.93* | 4.79 | 4.03* | 1.57 |
| ***MitoApo*** | 470 | 0.44* | 0.07 | 0.68* | 0.07 | 1.32* | 0.41 | 577.73 | 45.64 | 23.29* | 4.33 | 2.27* | 0.61 |
| ***Formulas in Supplemental Table 7*** | | 8r | | - | | 8s | | 8t | | 8u | | 8v | |

One-way ANOVA, n=3-7 fields per group, *p<0.05 as indicated by a Tukey’s comparison test to the control. Ave = Average, and S.D. = Standard Deviation.

**Supplementary Table 4:** Proteostat, mt-GFP, and p62 quantification of MDA-MB-231 cells based on area per cell.

| **Analyses of mt-GFP expressing MDA-MB-231 cells stained for p62 and aggregates** | | | | | | | | | | | | | |
| --- | --- | --- | --- | --- | --- | --- | --- | --- | --- | --- | --- | --- | --- |
|  | ***Cell Count*** | ***Pearson's correlation  for mt-GFP and Proteostat punctae (R value)*** | | ***Pearson's correlation  for p62 and mt-GFP (R value)*** | | ***Pearson's correlation  for p62 and Proteostat Punctae (R value)*** | | ***% area of  mt-GFP*** | | ***% area of  Proteostat punctae*** | | ***% area of p62*** | |
|  |  |  |  |  |  |  |  |  |  |  |  |  |  |
| ***Treatment*** |  | Ave. | S.D. | Ave. | S.D. | Ave. | S.D. | Ave. | S.D. | Ave. | S.D. | Ave. | S.D. |
| ***Control*** | 449 | 0.37 | 0.16 | 0.31 | 0.19 | 0.13 | 0.11 | 18.29 | 1.83 | 0.43 | 0.27 | 0.02 | 0.03 |
| ***CCCP*** | 367 | 0.81* | 0.11 | 0.5038 | 0.19 | 0.17 | 0.09 | 18.41 | 1.29 | 4.72* | 0.97 | 2.69 | 1.15 |
| ***MitoQ*** | 656 | 0.90* | 0.02 | 0.22 | 0.17 | 0.11 | 0.19 | 26.08 | 2.15 | 8.56* | 0.92 | 0.14 | 0.07 |
| ***MitoApo*** | 484 | 0.89* | 0.02 | 0.18 | 0.07 | 0.08 | 0.06 | 37.92 | 10.14 | 14.79* | 2.21 | 0.63 | 0.37 |
| ***Formulas in Supplemental Table 7*** | | - | | - | | - | | 8o | | 8h | | 8ff | |
|  | | ***% Proteostat area in mitochondria*** | | ***% mitochondrial area with Proteostat*** | | ***% p62 area in mitochondria*** | | ***% mitochondria  area with  p62*** | | ***% p62 area  in mitochondrial  Proteostat*** | |  | |
| ***Treatment*** | | Ave. | S.D. | Ave. | S.D. | Ave. | S.D. | Ave. | S.D. | Ave. | S.D. |  |  |
| ***Control*** | | 95.58 | 3.25 | 2.52 | 1.45 | 63.58 | 33.34 | 0.11 | 0.1 | 2.88 | 4.07 |  |  |
| ***CCCP*** | | 97.85 | 4.35 | 25.41 | 3.62 | 10.95 | 6.08 | 1.71 | 1.15 | 57.89 | 17.69 |  |  |
| ***MitoQ*** | | 96.52 | 3.25 | 25.21 | 4.25 | 19.57 | 5.14 | 0.07 | 0.03 | 45.47 | 19.68 |  |  |
| ***MitoApo*** | | 98.22 | 1.22 | 32.05 | 8.95 | 10.75 | 4.69 | 0.13 | 0.08 | 33.45 | 17.01 |  |  |
| ***Formulas in Supplemental Table 7*** | | 8p | | 8x | | 8gg | | 8hh | | 8ii | |  |  |

One-way ANOVA, n=5-6 fields per group, *p<0.05 as indicated by a Tukey’s comparison test to the control. Ave = Average, and S.D. = Standard Deviation.

**Supplementary Table 5:** Proteostat, mt-GFP, and TAX1BP1 quantification of MDA-MB-231 cells based on area per cell.

| **Analyses of mt-GFP expressing MDA-MB-231 cells stained for TAX1-BP1 and aggregates** | | | | | | | | | | | | | |
| --- | --- | --- | --- | --- | --- | --- | --- | --- | --- | --- | --- | --- | --- |
|  | ***Cell Count*** | ***Pearson's correlation   for mt-GFP and Proteostat punctae  (R value)*** | | ***Pearson's Correlation   for TAX1-BP1 and mt-GFP  (R value)*** | | ***Pearson's Correlation   for TAX1-BP1 and Proteostat punctae  (R value)*** | | ***% area of  mt-GFP*** | | ***% area of  Proteostat punctae*** | | ***% area of TAX1BP1*** | |
|  |  |  |  |  |  |  |  |  |  |  |  |  |  |
| ***Treatment*** |  | Ave. | S.D. | Ave. | S.D. | Ave. | S.D. | Ave. | S.D. | Ave. | S.D. | Ave. | S.D. |
| ***Control*** | 490 | 0.14 | 0.06 | 0.28 | 0.16 | 0.19 | 0.05 | 17.69 | 2.95 | 1.8 | 1.2 | 10.43 | 1.08 |
| ***CCCP*** | 649 | 0.6* | 0.13 | 0.22 | 0.06 | 0.18 | 0.07 | 19.96 | 2.32 | 7.36* | 0.88 | 12.7 | 1.21 |
| ***MitoQ*** | 849 | 0.72* | 0.02 | 0.17 | 0.08 | 0.17 | 0.07 | 15.26 | 1.59 | 4.28* | 0.47 | 7.26 | 0.86 |
| ***MitoApo*** | 492 | 0.71* | 0.17 | 0.17 | 0.11 | 0.21 | 0.07 | 12.43 | 4.43 | 3.51* | 0.57 | 18.33 | 6.02 |
| ***Formulas in Supplemental Table 7*** | | - | | - | | - | | 8o | | 8h | | 8w | |
|  | | ***% of Proteostat area in mt-GFP*** | | ***% mt-GFP area  with Proteostat*** | | ***% TAX1-BP1 area with mt-GFP*** | | ***% mt-GFP area with  TAX1-BP1*** | | ***% TAX1-BP1 area in mitochondrial  Proteostat*** | |  | |
| ***Treatment*** | | Ave. | S.D. | Ave. | S.D. | Ave. | S.D. | Ave. | S.D. | Ave. | S.D. |  |  |
| ***Control*** | | 86.23 | 8.32 | 1.11 | 0.6 | 39.42 | 18.74 | 60.72 | 18.92 | 1.8 | 1.8 |  |  |
| ***CCCP*** | | 89.03 | 23.37 | 32.96* | 9.25 | 59.68 | 6.98 | 37.87* | 12.71 | 25.02* | 3.53 |  |  |
| ***MitoQ*** | | 97.67 | 1.46 | 24.87* | 8.9 | 48.92 | 5.73 | 23.78* | 6.87 | 7.15* | 1.62 |  |  |
| ***MitoApo*** | | 87.26 | 6.12 | 22.32* | 7.51 | 31.72 | 9.52 | 25.39* | 6.29 | 5.9* | 1.26 |  |  |
| ***Formulas in Supplemental Table 7*** | | 8p | | 8x | | 8y | | 8z | | 8aa | |  |  |

One-way ANOVA, n=4-5 fields per group, *p<0.05 as indicated by a Tukey’s comparison test to the control. Ave = Average, and S.D. = Standard Deviation.

**Supplementary Table 6:** Proteostat, mt-GFP, and NDP52 quantification of MDA-MB-231 cells based on area per cell.

| **Analyses of mt-GFP expressing MDA-MB-231 cells stained for NDP52 and aggregates** | | | | | | | | | | | | | |
| --- | --- | --- | --- | --- | --- | --- | --- | --- | --- | --- | --- | --- | --- |
|  | ***Cell Count*** | ***Pearson's correlation  for mt-GFP and Proteostat punctae (R value)*** | | ***Pearson's correlation  for NDP52 and mt-GFP (R value)*** | | ***Pearson's correlation  for NDP52 and Proteostat Punctae (R value)*** | | ***% area of  mt-GFP*** | | ***% area of  Proteostat punctae*** | | ***% area of NDP52*** | |
|  |  |  |  |  |  |  |  |  |  |  |  |  |  |
| ***Treatment*** |  | Ave. | S.D. | Ave. | S.D. | Ave. | S.D. | Ave. | S.D. | Ave. | S.D. | Ave. | S.D. |
| ***Control*** | 456 | 0.15 | 0.1 | 0.21 | 0.16 | 0.14 | 0.06 | 10.5 | 0.1 | 0.06 | 0.04 | 23.54 | 0.93 |
| ***CCCP*** | 505 | 0.91* | 0.03 | 0.47 | 0.22 | 0.49* | 0.05 | 11.01 | 0.79 | 1.2* | 0.34 | 19.9 | 2.77 |
| ***MitoQ*** | 472 | 0.82* | 0.07 | 0.16 | 0.17 | 0.45* | 0.14 | 11.11 | 0.81 | 1.49* | 0.33 | 30.58 | 2.44 |
| ***MitoApo*** | 419 | 0.69* | 0.12 | 0.16 | 0.09 | 0.53* | 0.17 | 18.59 | 11.68 | 2.74* | 0.43 | 24.39 | 3.45 |
| ***Formulas in Supplemental Table 7*** | | - | | - | | - | | 8o | | 8h | | 8bb | |
|  | | ***% Proteostat area in mitochondria*** | | ***% mitochondrial area with Proteostat*** | | ***% NDP52 area in mitochondria*** | | ***% mitochondria  area with  NDP52*** | | ***% NDP52 area  in mitochondrial  Proteostat*** | |  | |
| ***Treatment*** | | Ave. | S.D. | Ave. | S.D. | Ave. | S.D. | Ave. | S.D. | Ave. | S.D. |  |  |
| ***Control*** | | 84.59 | 18.71 | 0.44 | 0.28 | 24.55 | 5.42 | 54.36 | 7.16 | 0.02 | 0.02 |  |  |
| ***CCCP*** | | 88.72 | 6.87 | 9.64 | 1.86 | 31.68 | 4.36 | 56.61 | 3.35 | 4.62* | 1.29 |  |  |
| ***MitoQ*** | | 89.42 | 4.48 | 3.74 | 2.49 | 28.44 | 4.64 | 64.43 | 14.77 | 1.89* | 0.5 |  |  |
| ***MitoApo*** | | 99.91 | 0.19 | 8.71 | 1.2 | 31.35 | 11.2 | 55.11 | 11.36 | 2.47* | 1.12 |  |  |
| ***Formulas in Supplemental Table 7*** | | 8p | | 8x | | 8cc | | 8dd | | 8ee | |  |  |

One-way ANOVA, n=5-6 fields per group, *p<0.05 as indicated by a Tukey’s comparison test to the control. Ave = Average, and S.D. = Standard Deviation.

**Supplementary Table 7:** Formulas used for confocal analyses.

| **Calculations for confocal imaging analyses** | |
| --- | --- |
| ***ID*** | ***Formulas*** |
| a | Total of positive cells with colocalized Proteostat and LC3 punctae / Total number of cells |
| b | Total number of LC3 punctae / Total number of cells |
| c | Total number of Proteostat punctae / Total number of cells |
| d | Number of LC3 punctae with Proteostat / Total number LC3 punctae |
| e | Number of Proteostat punctae without LC3 / Total number of Proteostat punctae |
| f | Total Area / Cell number |
| g | Total area of LC3 punctae / Total cellular area |
| h | Total area of Proteostat punctae / Total cellular area |
| i | Total area of LC3 without Proteostat / Total number of LC3 punctae without Proteostat |
| j | Total area of LC3 with Proteostat / Total number of LC3 punctae with Proteostat |
| k | Total of positive cells with colocalized Proteostat and LAMP1 punctae / Total number of cells |
| l | Total area of LAMP1 / Total cellular area |
| m | Total area of LAMP1 with Proteostat / Total area of Proteostat |
| n | Positive cells with colocalized Proteostat and mt-GFP Punctae / Total number of cells |
| o | Total area of mt-GFP / Total cellular area |
| p | Total Area of Proteostat with mt-GFP / Total Proteostat area |
| q | Total area of mitochondria - mt-GFP area with Proteostat / Total area of mitochondria |
| r | Total of positive cells with colocalized Proteostat and p53 punctae / Total number of cells |
| s | Number of Proteostat punctae with p53 / Total number of cells |
| t | Total area of p53 / Total cellular number of cells |
| u | Total area of Proteostat punctae / Total number of cells |
| v | Area of p53 with Proteostat / Total p53 area |
| w | Total area of TAX1BP1 / Total cellular area |
| x | Area of mt-GFP with Proteostat / Total area of mt-GFP |
| y | Area of Tax1BP1 with mt-GFP / Total area of TAX1BP1 |
| z | Area of TAX1BP1 with mt-GFP / Total area of mt-GFP |
| aa | Area of TAX1BP1 with Proteostat-labeled mt-GFP / Total area of Proteostat-labeled mt-GFP |
| bb | Total area of NDP52 / Total cellular area |
| cc | Area of NDP52 with mt-GFP / Total area of NDP52 |
| dd | Area of NDP52 with mt-GFP / Total area of mt-GFP |
| ee | Area of NDP52 with Proteostat-labeled mt-GFP / Total area of Proteostat-labeled mt-GFP |
| ff | Total area of p62 / Total cellular area |
| gg | Area of p62 with mt-GFP / Total area of NDP52 |
| hh | Area of p62 with mt-GFP / Total area of mt-GFP |
| ii | Area of p62 with Proteostat-labeled mt-GFP / Total area of Proteostat-labeled mt-GFP |

**Supplementary Table 8:** Parameters, figure locations, factors, degrees of freedom, and F and p values for the main results of two-way ANOVA analyses.

| **Two-Way ANOVA Analyses** | | | | |
| --- | --- | --- | --- | --- |
| Parameter | Figure | Factors | F (DFn, DFd) | p value |
| Aggregate Propensity Factor (APF) | Figure 1A | Interaction | F (12, 60) = 4.235 | P < 0.0001 |
|  |  | Cell type | F (3, 60) = 23.59 | P < 0.0001 |
|  |  | Treatment | F (4, 60) = 50.49 | P < 0.0001 |
| Ubiquitin and Proteostat cell population | Figure 1C | Interaction | F (12, 40) = 63.67 | P < 0.0001 |
|  |  | Quadrant | F (3, 40) = 1060 | P < 0.0001 |
|  |  | Treatment | F (4, 40) = 0.1540 | P = 0.9601 |
| Aggrephagy flux | Supplementary | Interaction | F (7, 80) = 2.371 | P = 0.0296 |
|  | Figure 1C | Treatments | F (7, 80) = 45.99 | P < 0.0001 |
|  |  | Bafilomycin | F (1, 80) = 9.034 | P = 0.0035 |
| GFP-LC3 with or without Proteostat | Figure 1J and | Interaction | F (3, 2792) = 329.3 | P < 0.0001 |
|  | Supplementary | Treatment | F (3, 2792) = 268.4 | P < 0.0001 |
|  | Table 1 | Proteostat | F (1, 2792) = 3139 | P < 0.0001 |
| APF in presence and absence of Bafilomycin | Figure 2D | Interaction | F (15, 96) = 2.935 | P = 0.0007 |
|  |  | Treatment | F (15, 96) = 22.93 | P < 0.0001 |
|  |  | Bafilomycin | F (1, 96) = 45.44 | P < 0.0001 |
| p53 levels | Figure 2I | Interaction | F (3, 16) = 0.2179 | P = 0.8826 |
|  |  | Treatment | F (3, 16) = 2.332 | P = 0.1129 |
|  |  | Bafilomycin | F (1, 16) = 0.1689 | P = 0.6865 |
| p53 levels | Figure 2H | Interaction | F (3, 16) = 1.617 | P = 0.2248 |
|  |  | Treatment | F (3, 16) = 27.60 | P < 0.0001 |
|  |  | Bafilomycin | F (1, 16) = 13.89 | P = 0.0018 |
| Lysotracker staining (Time as a Repeated Measure) | Supplementary | Interaction | F (6, 27) = 80.69 | P < 0.0001 |
|  | Figure 2B | Time | F (3, 27) = 290.4 | P < 0.0001 |
|  |  | Treatment | F (2, 9) = 215.5 | P < 0.0001 |
| Aggregate Propensity Factor (APF) | Supplementary | Interaction | F (2, 12) = 2.413 | P = 0.1315 |
|  | Figure 3C | Treatment | F (2, 12) = 84.22 | P < 0.0001 |
|  |  | ATG7 | F (1, 12) = 17.40 | P = 0.0013 |
| TAX1BP1 levels in MDA-MB-231 cells | Figure 3B | Interaction | F (2, 18) = 1.619 | P = 0.2257 |
|  |  | Treatment | F (2, 18) = 11.46 | P = 0.0006 |
|  |  | Bafilomycin | F (1, 18) = 100.8 | P < 0.0001 |
| TAX1BP1 levels in MCF-12A cells | Figure 3B | Interaction | F (2, 12) = 0.9219 | P = 0.4242 |
|  |  | Treatment | F (2, 12) = 0.5315 | P = 0.6009 |
|  |  | Bafilomycin | F (1, 12) = 82.21 | P < 0.0001 |
| TAX1BP1 levels in MCF-7 cells | Figure 3B | Interaction | F (2, 12) = 3.009 | P = 0.0873 |
|  |  | Treatment | F (2, 12) = 8.672 | P = 0.0047 |
|  |  | Bafilomycin | F (1, 12) = 32.55 | P < 0.0001 |
| TAX1BP1 levels in SKBR3 cells | Figure 3B | Interaction | F (2, 12) = 0.5216 | P = 0.6064 |
|  |  | Treatment | F (2, 12) = 0.3709 | P = 0.6978 |
|  |  | Bafilomycin | F (1, 12) = 59.27 | P < 0.0001 |
| NDP52 levels in MDA-MB-231 cells | Figure 3B | Interaction | F (2, 12) = 23.29 | P < 0.0001 |
|  |  | Treatment | F (2, 12) = 140.8 | P < 0.0001 |
|  |  | Bafilomycin | F (1, 12) = 430.8 | P < 0.0001 |
| NDP52 levels in MCF-12A cells | Figure 3B | Interaction | F (2, 12) = 0.9310 | P = 0.4209 |
|  |  | Treatment | F (2, 12) = 1.830 | P = 0.2024 |
|  |  | Bafilomycin | F (1, 12) = 34.33 | P < 0.0001 |
| NDP52 levels in MCF-7 cells | Figure 3B | Interaction | F (2, 12) = 0.1071 | P = 0.8992 |
|  |  | Treatment | F (2, 12) = 2.296 | P = 0.1431 |
|  |  | Bafilomycin | F (1, 12) = 17.77 | P = 0.0012 |
| NDP52 levels in SKBR3 cells | Figure 3B | Interaction | F (2, 12) = 0.1779 | P = 0.8392 |
|  |  | Treatment | F (2, 12) = 0.4356 | P = 0.6567 |
|  |  | Bafilomycin | F (1, 12) = 25.52 | P = 0.0003 |
| TAX1BP1 and NDP52 Levels | Figure 4A | Interaction | F (1, 12) = 69.48 | P < 0.0001 |
|  |  | SiRNA | F (1, 12) = 69.48 | P < 0.0001 |
|  |  | Protein | F (1, 12) = 0.4509 | P = 0.5146 |
| TAX1BP1 Levels | Figure 4B | Interaction | F (2, 12) = 0.1388 | P = 0.8718 |
|  |  | Treatment | F (2, 12) = 4.454 | P = 0.0357 |
|  |  | Knockdown | F (1, 12) = 31.46 | P = 0.0001 |
| NDP52 levels | Figure 4B | Interaction | F (2, 18) = 3.508 | P = 0.0517 |
|  |  | Treatment | F (2, 18) = 19.88 | P < 0.0001 |
|  |  | Knockdown | F (1, 18) = 28.41 | P < 0.0001 |
| TAX1BP1 levels | Figure 4C | Interaction | F (1, 12) = 0.9476 | P = 0.3495 |
|  |  | Treatment | F (1, 12) = 112.4 | P < 0.0001 |
|  |  | Knockdown | F (1, 12) = 181.8 | P < 0.0001 |
| NDP52 levels | Figure 4C | Interaction | F (1, 12) = 8.912 | P = 0.0114 |
|  |  | Treatment | F (1, 12) = 13.69 | P = 0.0030 |
|  |  | Knockdown | F (1, 12) = 14.00 | P = 0.0028 |
| LC3-II levels | Figure 4C | Interaction | F (3, 32) = 5.379 | P = 0.0041 |
|  |  | Treatment | F (3, 32) = 19.55 | P < 0.0001 |
|  |  | Knockdown | F (1, 32) = 117.6 | P < 0.0001 |
| TAX1BP1 Levels | Figure 4E | Interaction | F (2, 18) = 0.7774 | P = 0.4744 |
|  |  | Treatment | F (2, 18) = 7.919 | P = 0.0034 |
|  |  | Knockdown | F (1, 18) = 17.31 | P = 0.0006 |
| NDP52 levels | Figure 4D | Interaction | F (2, 18) = 0.4120 | P = 0.6684 |
|  |  | Treatment | F (2, 18) = 6.692 | P = 0.0067 |
|  |  | Knockdown | F (1, 18) = 16.78 | P = 0.0007 |
| LC3-II levels (MitoQ Treatment) | Figure 4F | Interaction | F (3, 32) = 0.7743 | P = 0.5170 |
|  |  | Treatment | F (3, 32) = 4.922 | P = 0.0064 |
|  |  | Knockdown | F (1, 32) = 23.64 | P < 0.0001 |
| LC3-II levels (MitoApo Treatment) | Figure 4 F | Interaction | F (3, 32) = 4.477 | P = 0.0098 |
|  |  | Baf | F (3, 32) = 14.77 | P < 0.0001 |
|  |  | Knockdown | F (1, 32) = 93.98 | P < 0.0001 |
| Aggregates in knockdowns | Figure 5A | Interaction | F (9, 57) = 1.798 | P = 0.0885 |
|  |  | Treatment | F (3, 57) = 31.90 | P < 0.0001 |
|  |  | Knockdown | F (3, 57) = 5.009 | P = 0.0038 |
| Mitochondrial delivery to lysosome | Figure 5B | Interaction | F (3, 16) = 1.773 | P = 0.1928 |
|  |  | Knockdown | F (3, 16) = 3.788 | P = 0.0316 |
|  |  | Bafilomycin | F (1, 16) = 34.30 | P < 0.0001 |
| Mitochondrial delivery to lysosome | Figure 5C | Interaction | F (3, 16) = 0.3775 | P = 0.7705 |
|  |  | Knockdown | F (3, 16) = 4.442 | P = 0.0188 |
|  |  | Bafilomycin | F (1, 16) = 486.5 | P < 0.0001 |
| Mitochondrial delivery to lysosome | Figure 5D | Interaction | F (3, 16) = 0.4192 | P = 0.7417 |
|  |  | Knockdown | F (3, 16) = 1.310 | P = 0.3056 |
|  |  | Bafilomycin | F (1, 16) = 110.6 | P < 0.0001 |
| Cell Death | Figure 5E | Interaction | F (9, 32) = 0.8519 | P = 0.5757 |
|  |  | Knockdown | F (3, 32) = 15.67 | P < 0.0001 |
|  |  | Treatment | F (3, 32) = 66.35 | P < 0.0001 |

**Supplementary Table 9:** Parameters, figure locations, factors, degrees of freedom, and F and p values for the main results of one-way ANOVA analyses.

| **Ordinary ANOVA Analyses** | | | |
| --- | --- | --- | --- |
| Parameter | Figure location | F (DFn, DFd) | p value |
| 1 mM> x < 5 mM Particles in Cell lysate | Figure 1E | F (4, 30) = 5.984 | P = 0.0012 |
| Pearson Correlation GFP-LC3 and Proteostat Punctae | Figure 2a and Supplementary Table 1 | F (3, 9) = 23.80 | P = 0.0001 |
| Population GFP-LC3 and Proteostat Punctae | Figure 2a and Supplementary Table 1 | F (3, 25) = 70.88 | P < 0.001 |
| GFP-LC3 Proteostat Punctae | Figure 1G and Supplementary Table 1 | F (3, 9) = 14.56 | P = 0.0008 |
| Proteostat Punctae | Figure 1G and Supplementary Table 1 | F (3, 9) = 25.87 | P < 0.0001 |
| GFP-LC3 colocalized with Proteostat Punctae | Figure 1I and Supplementary Table 1 | F (3, 9) = 18.64 | P = 0.0003 |
| Proteostat not localized with GFP-LC3 (Area) | Supplementary Table 1 | F (3, 9) = 1.955 | P = 0.1914 |
| Cell Size (Area) | Supplementary Table 1 | F (3, 9) = 8.541 | P = 0.0053 |
| GFP-LC3 Punctae (Area) | Figure 1H and Supplementary Table 1 | F (3, 9) = 9.435 | P = 0.0039 |
| Proteostat Punctae (Area) | Figure 1H and Supplementary Table 1 | F (3, 9) = 6.590 | P = 0.0119 |
| LAMP1- and Proteostat- stained total cell area | Supplementary Table 2 | F (3, 25) = 4.571 | P = 0.0110 |
| Pearson Correlation Lamp1 and Proteostat Punctae | Figure 2A and Supplementary Table 2 | F (3, 9) = 18.90 | P = 0.0003 |
| Cell population with Proteostat punctae with LAMP1 | Figure 2A and Supplementary Table 2 | F (3, 21) = 39.94 | P < 0.0001 |
| LAMP1/Total Cell Area | Figure 2B and Supplementary Table 2 | F (3, 24) = 6.713 | P = 0.0019 |
| Proteostat/Total Cell Area | Figure 2B and Supplementary Table 2 | F (3, 24) = 17.30 | P < 0.0001 |
| LAMP1 Area with Proteostat | Figure 2C | F (3, 24) = 77.19 | P < 0.0001 |
| LAMP1 Levels | Figure 2F | F (4, 20) = 13.55 | P < 0.0001 |
| Cathepsin Levels | Figure 2F | F (4, 10) = 12.56 | P = 0.0007 |
| LAL Levels | Figure 2F | F (4, 25) = 36.69 | P < 0.0001 |
| % of Population positive for p53 and Proteostat colocalization | Figure 2G and Supplementary Table 4 | F (3, 17) = 59.51 | P < 0.0001 |
| Pearson Correlation for p53 and Proteostat | Figure 2G and Supplementary Table 3 | F (3, 8) = 45.62 | P < 0.0001 |
| p53 and Proteostat Punctae per cell | Supplementary Table 3 | F (3, 17) = 51.65 | P < 0.0001 |
| Area of Proteostat Punctae per cell | Supplementary Table 3 | F (3, 17) = 85.08 | P < 0.0001 |
| Proteostat in p53 Area | Supplementary Table 3 | F (3, 17) = 43.75 | P < 0.0001 |
| Protein stability - OPTN Levels at 24 hours | Supplementary Figure 3A | F (3, 12) = 7.124 | P = 0.0053 |
| Protein stability - TAX1BP1 Levels at 24 hours | Supplementary Figure 3A | F (3, 8) = 98.23 | P < 0.0001 |
| Protein stability - p62 Levels at 24 hours | Supplementary Figure 3A | F (3, 12) = 3.373 | P = 0.0546 |
| Protein stability - NDP52 Levels at 24 hours | Supplementary Figure 3A | F (3, 8) = 52.72 | P < 0.0001 |
| Degradation mechanisms - OPTN Levels at 24 hours | Figure 3A - MitoQ | F (3, 12) = 22.25 | P < 0.0001 |
| Degradation mechanisms - TAX1BP1 Levels at 24 hours | Figure 3A - MitoQ | F (3, 16) = 11.41 | P = 0.0003 |
| Degradation mechanisms - p62 Levels at 24 hours | Figure 3A - MitoQ | F (3, 16) = 2.938 | P = 0.0650 |
| Degradation mechanisms - NDP52 Levels at 24 hours | Figure 3A - MitoQ | F (3, 11) = 57.63 | P < 0.0001 |
| Degradation mechanisms - OPTN Levels at 24 hours | Figure 3A - MitoApo | F (3, 8) = 13.99 | P = 0.0015 |
| Degradation mechanisms - TAX1BP1 Levels at 24 hours | Figure 3A - MitoApo | F (3, 8) = 38.00 | P < 0.0001 |
| Degradation mechanisms - p62 Levels at 24 hours | Figure 3A - MitoApo | F (3, 12) = 1.626 | P = 0.2354 |
| Degradation mechanisms - NDP52 Levels at 24 hours | Figure 3A - MitoApo | F (3, 8) = 49.81 | P < 0.0001 |
| Pearson Correlation for mt-GFP and Proteostat in whole cells - p62 stained cells | Supplementary Figure 4D and Supplementary Table 4 | F (3, 17) = 72.99 | P < 0.0001 |
| Pearson Correlation for mt-GFP and Proteostat in whole cells -TAX1BP1 stained cells | Figure 3C and Supplementary Table 5 | F (3, 15) = 32.14 | P < 0.0001 |
| Pearson Correlation for mt-GFP and Proteostat in whole cells - NDP52 stained cells | Figure 3D and Supplementary Table 6 | F (3, 17) = 24.39 | P < 0.0001 |
| Pearson Correlation for mt-GFP and p62 in whole cells - p62 stained cells | Supplementary Figure 4D and Supplementary Table 4 | F (3, 17) = 4.213 | P = 0.0212 |
| Pearson Correlation for mt-GFP and TAX1BP1 in whole cells -TAX1BP1 stained cells | Figure 3C and Supplementary Table 5 | F (3, 15) = 1.100 | P = 0.3798 |
| Pearson Correlation for mt-GFP and NDP52 in whole cells - NDP52 stained cells | Figure 3D and Supplementary Table 6 | F (3, 17) = 5.098 | P = 0.0107 |
| Pearson Correlation for Proteostat and p62 in whole cells - p62 stained cells | Supplementary Figure 4D and Supplementary Table 4 | F (3, 17) = 1.047 | P = 0.3972 |
| Pearson Correlation for Proteostat and TAX1BP1 in whole cells -TAX1BP1 stained cells | Figure 3C and Supplementary Table 5 | F (3, 15) = 0.3006 | P = 0.8244 |
| Pearson Correlation for Proteostat and NDP52 in whole cells - NDP52 stained cells | Figure 3D and Supplementary Table 6 | F (3, 17) = 11.25 | P = 0.0003 |
| Pearson Correlation for Proteostat-labeled mt-GFP and p62 in whole cells - p62 stained cells | Supplementary Figure 4D and Supplementary Table 4 | F (3, 17) = 12.12 | P = 0.0002 |
| Pearson Correlation for Proteostat-labeled mt-GFP and TAX1BP1 in whole cells -TAX1BP1 stained cells | Figure 3C and Supplementary Table 5 | F (3, 15) = 100.7 | P < 0.0001 |
| Pearson Correlation for Proteostat-labeled mt-GFP and NDP52 in whole cells - NDP52 stained cells | Figure 3D and Supplementary Table 6 | F (3, 17) = 1.050 | P = 0.3959 |
| % of area of Proteostat Punctae - p62 stained cells | Supplementary Table 4 | F (3, 17) = 114.2 | P < 0.0001 |
| % of area of Proteostat Punctae - TAX1BP1 stained cells | Supplementary Table 5 | F (3, 17) = 61.85 | P < 0.0001 |
| % of area of Proteostat Punctae - NDP52 stained cells | Supplementary Table 6 | F (3, 16) = 39.18 | P < 0.0001 |
| Percentage of mt-GFP area with overlapping p62 | Supplementary Table 4 | F (3, 17) = 9.390 | P = 0.0007 |
| Percentage of mt-GFP area with overlapping TAX1BP1 | Supplementary Table 5 | F (3, 16) = 9.599 | P = 0.0007 |
| Percentage of mt-GFP area with overlapping NDP52 | Supplementary Table 6 | F (3, 17) = 1.050 | P = 0.3959 |
| Percentage of p62 area with overlapping Proteostat-labeled mt-GFP | Supplementary Table 4 | F (3, 17) = 11.71 | P = 0.0002 |
| Percentage of TAX1BP1 area with overlapping Proteostat-labeled mt-GFP | Supplementary Table 5 | F (3, 15) = 5.013 | P = 0.0133 |
| Percentage of NDP52 area with overlapping Proteostat-labeled mt-GFP | Supplementary Table 6 | F (3, 17) = 1.058 | P = 0.3927 |
| Percentage of p62 area with overlapping Proteostat | Supplementary Table 4 | F (3, 17) = 12.12 | P = 0.0002 |
| Percentage of TAX1BP1 area with overlapping Proteostat | Supplementary Table 5 | F (3, 15) = 100.7 | P < 0.0001 |
| Percentage of MDP52 area with overlapping Proteostat | Supplementary Table 6 | F (3, 17) = 21.92 | P < 0.0001 |

**Supplementary Table 10:** Parameters, figure locations, degrees of freedom, and p values for Student’s T tests.

| Student T Tests | | | |
| --- | --- | --- | --- |
| Parameter | Figure location | DF | p Value |
| PINK1 levels | Supplementary Figure 5A | 4 | 0.0019 |
| Cell Death | Supplementary  Figure 5B | 4 | 0.00015 |
| p53 levels | Figure 6B | 12 | 0.04729 |
| LC3-II levels | Figure 6B | 12 | 0.20765 |
| p62 levels | Figure 6B | 12 | 0.00740 |
| OPTN levels | Figure 6B | 12 | 0.01195 |
| NDP52 levels | Figure 6B | 12 | 0.00580 |
| LC3-II levels | Figure 6C | 4 | 0.01293 |
| TAX1BP1 levels | Figure 6C | 4 | 0.82457 |
| p62 levels | Figure 6C | 4 | 0.44908 |
| OPTN levels | Figure 6C | 4 | 0.01824 |
| NDP52 levels | Figure 6C | 4 | 0.82848 |
