# Supplementary material for: Mitochondrial dysfunction generates aggregates that resist lysosomal degradation in human breast cancer cells
Source: Cell Death Dis. 2020 Jun 15;11(6):460. doi: 10.1038/s41419-020-2658-y (PMC7296005; doi:10.1038/s41419-020-2658-y)
Supplement: Supplementary file 10 — Supplemental Table 2 [file 41419_2020_2658_MOESM10_ESM.docx]

**Supplementary Table 1:** LC3 and Proteostat punctae and area quantification in MDA-MB-231 cells based on the number of punctae per cell.

| **Punctae analyses of MDA-MB-231 cells stained for LC3 and aggregates** | | | | | | | | | | | | | |
| --- | --- | --- | --- | --- | --- | --- | --- | --- | --- | --- | --- | --- | --- |
|  | ***Cell Count*** | ***Pearson's Correlation (R values)*** | | ***Population %  positive for  colocalized punctae*** | | ***GFP-LC3 punctae  per cell*** | | ***Proteostat punctae  per cell*** | | ***% of GFP-LC3  punctae with Proteostat*** | | ***% of Proteostat punctae  without GFP-LC3*** | |
| ***Treatment*** |  | Ave. | S.D. | Ave. | S.D. | Ave. | S.D. | Ave. | S.D. | Ave. | S.D. | Ave. | S.D. |
| ***Control*** | 529 | 0.27 | 0.115 | 23 | 6.1 | 6.49 | 1.93 | 2.91 | 0.9 | 36.02 | 1.25 | 19.15 | 1.56 |
| ***CCCP*** | 481 | 0.56* | 0.062 | 53* | 7.3 | 5.33 | 1.82 | 3.71 | 0.37 | 63.15* | 4.4 | 10.83 | 9.88 |
| ***MitoQ*** | 461 | 0.74* | 0.023 | 67* | 5.8 | 13.85* | 2.29 | 9.34* | 1.37 | 60.85* | 5.45 | 9.74 | 8.12 |
| ***MitoApo*** | 583 | 0.65* | 0.065 | 62* | 6.6 | 9.83* | 0.87 | 6.79* | 1.36 | 64.71* | 8.7 | 5.9 | 0.65 |
| ***Formula in Supplemental. Table 7*** | | - | | 8a | | 8b | | 8c | | 8d | | 8e | |
| **Area analyses of MDA-MB-231 cells stained for LC3 and aggregates** | | | | | | | | | | | |  |  |
|  |  | ***Area per  cell(AC) (μm^2)*** | | ***% of area with GFP-LC3 punctae*** | | ***% of area with Proteostat punctae*** | | ***LC3 punctae (μm^2)*** | | | |  |  |
|  |  |  |  |  |  |  |  | ***without  Proteostat (n=350)*** | | ***with  Proteostat  (n = 350)*** | |  |  |
| ***Treatment*** | | Ave. | S.D. | Ave. | S.D. | Ave. | S.D. | Ave. | S.D. | Ave. | S.D. |  |  |
| ***Control*** | | 825.42 | 29.61 | 2.23 | 0.82 | 0.8 | 0.27 | 1.7 | 1.7 | 5.29# | 7.11 |  |  |
| ***CCCP*** | | 458.89* | 122.18 | 7.97* | 1.06 | 4.69* | 0.58 | 1.19 | 1.07 | 15.44#* | 11.71 |  |  |
| ***MitoQ*** | | 827.73 | 163.52 | 9.91* | 1.49 | 4.71* | 1.05 | 1.25 | 1.07 | 22.22#* | 12.38 |  |  |
| ***MitoApo*** | | 749.8 | 85.64 | 10.65* | 4.02 | 5.79* | 2.86 | 1.27 | 1.06 | 28.66#* | 18.91 |  |  |
| ***Formula in Supplemental Table 7*** | | 8f | | 8g | | 8h | | 8i | | 8j | |  |  |

One- and two-way ANOVA, n=4-7 fields per group, *p<0.05 as indicated by a Tukey’s comparison test to the control, while #p<0.05 between groups. Ave = Average, and S.D. = Standard Deviation.
