# Supplementary material for: Mitochondrial dysfunction generates aggregates that resist lysosomal degradation in human breast cancer cells
Source: Cell Death Dis. 2020 Jun 15;11(6):460. doi: 10.1038/s41419-020-2658-y (PMC7296005; doi:10.1038/s41419-020-2658-y)
Supplement: Supplementary file 11 — Supplemental Table 3 [file 41419_2020_2658_MOESM11_ESM.docx]

**Supplementary Table 2:** LAMP1 and Proteostat quantification in MDA-MB-231 cells based on area per cell.

| **Area analyses of MDA-MB-231 cells stained for LAMP1A and aggregates** | | | | | | | | | | | | | |
| --- | --- | --- | --- | --- | --- | --- | --- | --- | --- | --- | --- | --- | --- |
|  | ***Cell Count*** | ***Area  per cell (mm^2)*** | | ***Pearson's Correlation  (R values)*** | | ***Population %  positive for  colocalized punctae*** | | ***% of area with LAMP1*** | | ***% of area with Proteostat punctae*** | | ***% of Proteostat punctae within  LAMP1*** | |
|  |  |  |  |  |  |  |  |  |  |  |  |  |  |
| ***Treatment*** |  | Ave. | S.D. | Ave. | S.D. | Ave. | S.D. | Ave. | S.D. | Ave. | S.D. | Ave. | S.D. |
| ***Control*** | 415 | 873.17 | 29.21 | 0.17 | 0.07 | 23.59 | 11.53 | 12.74 | 3.14 | 1.36 | 0.56 | 96.43 | 3.23 |
| ***CCCP*** | 252 | 698.91* | 175.63 | 0.68* | 0.12 | 90.41* | 4.5 | 19.12* | 1.57 | 6.43* | 0.89 | 98.98 | 0.68 |
| ***MitoQ*** | 368 | 855.91 | 75.56 | 0.73* | 0.09 | 47.85* | 8.09 | 21.03* | 5.32 | 5.19* | 2.24 | 98.84 | 0.61 |
| ***MitoApo*** | 455 | 861.09 | 70.45 | 0.68* | 0.12 | 58.36* | 11.29 | 23.99* | 6.69 | 10.23* | \| 4.05 \| \| --- \| | 99.69 | 0.25 |
| ***Formulas in Supplemental Table 7*** | | 8f | | - | | 8k | | 8l | | 8h | | 8m | |

One-way ANOVA, n=4-8 fields per group, *p<0.05 as indicated by a Tukey’s comparison test to the control, while #p<0.05 between groups. Ave = Average, and S.D. = Standard Deviation.
