# Supplementary material for: Mitochondrial dysfunction generates aggregates that resist lysosomal degradation in human breast cancer cells
Source: Cell Death Dis. 2020 Jun 15;11(6):460. doi: 10.1038/s41419-020-2658-y (PMC7296005; doi:10.1038/s41419-020-2658-y)
Supplement: Supplementary file 12 — Supplemental Table 4 [file 41419_2020_2658_MOESM12_ESM.docx]

**Supplementary Table 3:** Proteostat and p53 quantification of MDA-MB-231 cells based on area per cell.

| **Punctae and area analyses of MDA-MB-231 cells stained for p53 and aggregates** | | | | | | | | | | | | | |
| --- | --- | --- | --- | --- | --- | --- | --- | --- | --- | --- | --- | --- | --- |
|  | ***Cell Count*** | ***% of cells positive for colocalization*** | | ***Pearson Correlation  (R values)*** | | ***Colocalized punctae per cell*** | | ***Area of  p53 per cell  (μm^2)*** | | ***Area of  Proteostat punctae per cell (μm^2)*** | | ***% of p53 area with  Proteostat*** | |
|  |  |  |  |  |  |  |  |  |  |  |  |  |  |
| ***Treatment*** |  | Ave. | S.D. | Ave. | S.D. | Ave. | S.D. | Ave. | S.D. | Ave. | S.D. | Ave. | S.D. |
| ***Control*** | 437 | 0.12 | 0.06 | 0.13 | 0.04 | 0.08 | 0.08 | 594.37 | 57.58 | 1.77 | 1.65 | 0.291 | 0.267 |
| ***CCCP*** | 588 | 0.83* | 0.053 | 0.67* | 0.06 | 4.87* | 0.98 | 695.36 | 76.88 | 65.21* | 10.88 | 9.41* | 1.42 |
| ***MitoQ*** | 383 | 0.59* | 0.07 | 0.69* | 0.08 | 2.64* | 0.49 | 677.46 | 49.65 | 30.93* | 4.79 | 4.03* | 1.57 |
| ***MitoApo*** | 470 | 0.44* | 0.07 | 0.68* | 0.07 | 1.32* | 0.41 | 577.73 | 45.64 | 23.29* | 4.33 | 2.27* | 0.61 |
| ***Formulas in Supplemental Table 7*** | | 8r | | - | | 8s | | 8t | | 8u | | 8v | |

One-way ANOVA, n=3-7 fields per group, *p<0.05 as indicated by a Tukey’s comparison test to the control. Ave = Average, and S.D. = Standard Deviation.
