# Supplementary material for: Mitochondrial dysfunction generates aggregates that resist lysosomal degradation in human breast cancer cells
Source: Cell Death Dis. 2020 Jun 15;11(6):460. doi: 10.1038/s41419-020-2658-y (PMC7296005; doi:10.1038/s41419-020-2658-y)
Supplement: Supplementary file 13 — Supplemental Table 5 [file 41419_2020_2658_MOESM13_ESM.docx]

**Supplementary Table 4:** Proteostat, mt-GFP, and p62 quantification of MDA-MB-231 cells based on area per cell.

| **Analyses of mt-GFP expressing MDA-MB-231 cells stained for p62 and aggregates** | | | | | | | | | | | | | |
| --- | --- | --- | --- | --- | --- | --- | --- | --- | --- | --- | --- | --- | --- |
|  | ***Cell Count*** | ***Pearson's correlation  for mt-GFP and Proteostat punctae (R value)*** | | ***Pearson's correlation  for p62 and mt-GFP (R value)*** | | ***Pearson's correlation  for p62 and Proteostat Punctae (R value)*** | | ***% area of  mt-GFP*** | | ***% area of  Proteostat punctae*** | | ***% area of p62*** | |
|  |  |  |  |  |  |  |  |  |  |  |  |  |  |
| ***Treatment*** |  | Ave. | S.D. | Ave. | S.D. | Ave. | S.D. | Ave. | S.D. | Ave. | S.D. | Ave. | S.D. |
| ***Control*** | 449 | 0.37 | 0.16 | 0.31 | 0.19 | 0.13 | 0.11 | 18.29 | 1.83 | 0.43 | 0.27 | 0.02 | 0.03 |
| ***CCCP*** | 367 | 0.81* | 0.11 | 0.5038 | 0.19 | 0.17 | 0.09 | 18.41 | 1.29 | 4.72* | 0.97 | 2.69 | 1.15 |
| ***MitoQ*** | 656 | 0.90* | 0.02 | 0.22 | 0.17 | 0.11 | 0.19 | 26.08 | 2.15 | 8.56* | 0.92 | 0.14 | 0.07 |
| ***MitoApo*** | 484 | 0.89* | 0.02 | 0.18 | 0.07 | 0.08 | 0.06 | 37.92 | 10.14 | 14.79* | 2.21 | 0.63 | 0.37 |
| ***Formulas in Supplemental Table 7*** | | - | | - | | - | | 8o | | 8h | | 8ff | |
|  | | ***% Proteostat area in mitochondria*** | | ***% mitochondrial area with Proteostat*** | | ***% p62 area in mitochondria*** | | ***% mitochondria  area with  p62*** | | ***% p62 area  in mitochondrial  Proteostat*** | |  | |
| ***Treatment*** | | Ave. | S.D. | Ave. | S.D. | Ave. | S.D. | Ave. | S.D. | Ave. | S.D. |  |  |
| ***Control*** | | 95.58 | 3.25 | 2.52 | 1.45 | 63.58 | 33.34 | 0.11 | 0.1 | 2.88 | 4.07 |  |  |
| ***CCCP*** | | 97.85 | 4.35 | 25.41 | 3.62 | 10.95 | 6.08 | 1.71 | 1.15 | 57.89 | 17.69 |  |  |
| ***MitoQ*** | | 96.52 | 3.25 | 25.21 | 4.25 | 19.57 | 5.14 | 0.07 | 0.03 | 45.47 | 19.68 |  |  |
| ***MitoApo*** | | 98.22 | 1.22 | 32.05 | 8.95 | 10.75 | 4.69 | 0.13 | 0.08 | 33.45 | 17.01 |  |  |
| ***Formulas in Supplemental Table 7*** | | 8p | | 8x | | 8gg | | 8hh | | 8ii | |  |  |

One-way ANOVA, n=5-6 fields per group, *p<0.05 as indicated by a Tukey’s comparison test to the control. Ave = Average, and S.D. = Standard Deviation.
