# Supplementary material for: Mitochondrial dysfunction generates aggregates that resist lysosomal degradation in human breast cancer cells
Source: Cell Death Dis. 2020 Jun 15;11(6):460. doi: 10.1038/s41419-020-2658-y (PMC7296005; doi:10.1038/s41419-020-2658-y)
Supplement: Supplementary file 14 — Supplemental Table 6 [file 41419_2020_2658_MOESM14_ESM.docx]

**Supplementary Table 5:** Proteostat, mt-GFP, and TAX1BP1 quantification of MDA-MB-231 cells based on area per cell.

| **Analyses of mt-GFP expressing MDA-MB-231 cells stained for TAX1-BP1 and aggregates** | | | | | | | | | | | | | |
| --- | --- | --- | --- | --- | --- | --- | --- | --- | --- | --- | --- | --- | --- |
|  | ***Cell Count*** | ***Pearson's correlation   for mt-GFP and Proteostat punctae  (R value)*** | | ***Pearson's Correlation   for TAX1-BP1 and mt-GFP  (R value)*** | | ***Pearson's Correlation   for TAX1-BP1 and Proteostat punctae  (R value)*** | | ***% area of  mt-GFP*** | | ***% area of  Proteostat punctae*** | | ***% area of TAX1BP1*** | |
|  |  |  |  |  |  |  |  |  |  |  |  |  |  |
| ***Treatment*** |  | Ave. | S.D. | Ave. | S.D. | Ave. | S.D. | Ave. | S.D. | Ave. | S.D. | Ave. | S.D. |
| ***Control*** | 490 | 0.14 | 0.06 | 0.28 | 0.16 | 0.19 | 0.05 | 17.69 | 2.95 | 1.8 | 1.2 | 10.43 | 1.08 |
| ***CCCP*** | 649 | 0.6* | 0.13 | 0.22 | 0.06 | 0.18 | 0.07 | 19.96 | 2.32 | 7.36* | 0.88 | 12.7 | 1.21 |
| ***MitoQ*** | 849 | 0.72* | 0.02 | 0.17 | 0.08 | 0.17 | 0.07 | 15.26 | 1.59 | 4.28* | 0.47 | 7.26 | 0.86 |
| ***MitoApo*** | 492 | 0.71* | 0.17 | 0.17 | 0.11 | 0.21 | 0.07 | 12.43 | 4.43 | 3.51* | 0.57 | 18.33 | 6.02 |
| ***Formulas in Supplemental Table 7*** | | - | | - | | - | | 8o | | 8h | | 8w | |
|  | | ***% of Proteostat area in mt-GFP*** | | ***% mt-GFP area  with Proteostat*** | | ***% TAX1-BP1 area with mt-GFP*** | | ***% mt-GFP area with  TAX1-BP1*** | | ***% TAX1-BP1 area in mitochondrial  Proteostat*** | |  | |
| ***Treatment*** | | Ave. | S.D. | Ave. | S.D. | Ave. | S.D. | Ave. | S.D. | Ave. | S.D. |  |  |
| ***Control*** | | 86.23 | 8.32 | 1.11 | 0.6 | 39.42 | 18.74 | 60.72 | 18.92 | 1.8 | 1.8 |  |  |
| ***CCCP*** | | 89.03 | 23.37 | 32.96* | 9.25 | 59.68 | 6.98 | 37.87* | 12.71 | 25.02* | 3.53 |  |  |
| ***MitoQ*** | | 97.67 | 1.46 | 24.87* | 8.9 | 48.92 | 5.73 | 23.78* | 6.87 | 7.15* | 1.62 |  |  |
| ***MitoApo*** | | 87.26 | 6.12 | 22.32* | 7.51 | 31.72 | 9.52 | 25.39* | 6.29 | 5.9* | 1.26 |  |  |
| ***Formulas in Supplemental Table 7*** | | 8p | | 8x | | 8y | | 8z | | 8aa | |  |  |

One-way ANOVA, n=4-5 fields per group, *p<0.05 as indicated by a Tukey’s comparison test to the control. Ave = Average, and S.D. = Standard Deviation.
