# Supplementary material for: Mitochondrial dysfunction generates aggregates that resist lysosomal degradation in human breast cancer cells
Source: Cell Death Dis. 2020 Jun 15;11(6):460. doi: 10.1038/s41419-020-2658-y (PMC7296005; doi:10.1038/s41419-020-2658-y)
Supplement: Supplementary file 15 — Supplemental Table 7 [file 41419_2020_2658_MOESM15_ESM.docx]

**Supplementary Table 6:** Proteostat, mt-GFP, and NDP52 quantification of MDA-MB-231 cells based on area per cell.

| **Analyses of mt-GFP expressing MDA-MB-231 cells stained for NDP52 and aggregates** | | | | | | | | | | | | | |
| --- | --- | --- | --- | --- | --- | --- | --- | --- | --- | --- | --- | --- | --- |
|  | ***Cell Count*** | ***Pearson's correlation  for mt-GFP and Proteostat punctae (R value)*** | | ***Pearson's correlation  for NDP52 and mt-GFP (R value)*** | | ***Pearson's correlation  for NDP52 and Proteostat Punctae (R value)*** | | ***% area of  mt-GFP*** | | ***% area of  Proteostat punctae*** | | ***% area of NDP52*** | |
|  |  |  |  |  |  |  |  |  |  |  |  |  |  |
| ***Treatment*** |  | Ave. | S.D. | Ave. | S.D. | Ave. | S.D. | Ave. | S.D. | Ave. | S.D. | Ave. | S.D. |
| ***Control*** | 456 | 0.15 | 0.1 | 0.21 | 0.16 | 0.14 | 0.06 | 10.5 | 0.1 | 0.06 | 0.04 | 23.54 | 0.93 |
| ***CCCP*** | 505 | 0.91* | 0.03 | 0.47 | 0.22 | 0.49* | 0.05 | 11.01 | 0.79 | 1.2* | 0.34 | 19.9 | 2.77 |
| ***MitoQ*** | 472 | 0.82* | 0.07 | 0.16 | 0.17 | 0.45* | 0.14 | 11.11 | 0.81 | 1.49* | 0.33 | 30.58 | 2.44 |
| ***MitoApo*** | 419 | 0.69* | 0.12 | 0.16 | 0.09 | 0.53* | 0.17 | 18.59 | 11.68 | 2.74* | 0.43 | 24.39 | 3.45 |
| ***Formulas in Supplemental Table 7*** | | - | | - | | - | | 8o | | 8h | | 8bb | |
|  | | ***% Proteostat area in mitochondria*** | | ***% mitochondrial area with Proteostat*** | | ***% NDP52 area in mitochondria*** | | ***% mitochondria  area with  NDP52*** | | ***% NDP52 area  in mitochondrial  Proteostat*** | |  | |
| ***Treatment*** | | Ave. | S.D. | Ave. | S.D. | Ave. | S.D. | Ave. | S.D. | Ave. | S.D. |  |  |
| ***Control*** | | 84.59 | 18.71 | 0.44 | 0.28 | 24.55 | 5.42 | 54.36 | 7.16 | 0.02 | 0.02 |  |  |
| ***CCCP*** | | 88.72 | 6.87 | 9.64 | 1.86 | 31.68 | 4.36 | 56.61 | 3.35 | 4.62* | 1.29 |  |  |
| ***MitoQ*** | | 89.42 | 4.48 | 3.74 | 2.49 | 28.44 | 4.64 | 64.43 | 14.77 | 1.89* | 0.5 |  |  |
| ***MitoApo*** | | 99.91 | 0.19 | 8.71 | 1.2 | 31.35 | 11.2 | 55.11 | 11.36 | 2.47* | 1.12 |  |  |
| ***Formulas in Supplemental Table 7*** | | 8p | | 8x | | 8cc | | 8dd | | 8ee | |  |  |

One-way ANOVA, n=5-6 fields per group, *p<0.05 as indicated by a Tukey’s comparison test to the control. Ave = Average, and S.D. = Standard Deviation.
