# Supplementary material for: Mitochondrial dysfunction generates aggregates that resist lysosomal degradation in human breast cancer cells
Source: Cell Death Dis. 2020 Jun 15;11(6):460. doi: 10.1038/s41419-020-2658-y (PMC7296005; doi:10.1038/s41419-020-2658-y)
Supplement: Supplementary file 16 — Supplemental Table 8 [file 41419_2020_2658_MOESM16_ESM.docx]

**Supplementary Table 7:** Formulas used for confocal analyses.

| **Calculations for confocal imaging analyses** | |
| --- | --- |
| ***ID*** | ***Formulas*** |
| a | Total of positive cells with colocalized Proteostat and LC3 punctae / Total number of cells |
| b | Total number of LC3 punctae / Total number of cells |
| c | Total number of Proteostat punctae / Total number of cells |
| d | Number of LC3 punctae with Proteostat / Total number LC3 punctae |
| e | Number of Proteostat punctae without LC3 / Total number of Proteostat punctae |
| f | Total Area / Cell number |
| g | Total area of LC3 punctae / Total cellular area |
| h | Total area of Proteostat punctae / Total cellular area |
| i | Total area of LC3 without Proteostat / Total number of LC3 punctae without Proteostat |
| j | Total area of LC3 with Proteostat / Total number of LC3 punctae with Proteostat |
| k | Total of positive cells with colocalized Proteostat and LAMP1 punctae / Total number of cells |
| l | Total area of LAMP1 / Total cellular area |
| m | Total area of LAMP1 with Proteostat / Total area of Proteostat |
| n | Positive cells with colocalized Proteostat and mt-GFP Punctae / Total number of cells |
| o | Total area of mt-GFP / Total cellular area |
| p | Total Area of Proteostat with mt-GFP / Total Proteostat area |
| q | Total area of mitochondria - mt-GFP area with Proteostat / Total area of mitochondria |
| r | Total of positive cells with colocalized Proteostat and p53 punctae / Total number of cells |
| s | Number of Proteostat punctae with p53 / Total number of cells |
| t | Total area of p53 / Total cellular number of cells |
| u | Total area of Proteostat punctae / Total number of cells |
| v | Area of p53 with Proteostat / Total p53 area |
| w | Total area of TAX1BP1 / Total cellular area |
| x | Area of mt-GFP with Proteostat / Total area of mt-GFP |
| y | Area of Tax1BP1 with mt-GFP / Total area of TAX1BP1 |
| z | Area of TAX1BP1 with mt-GFP / Total area of mt-GFP |
| aa | Area of TAX1BP1 with Proteostat-labeled mt-GFP / Total area of Proteostat-labeled mt-GFP |
| bb | Total area of NDP52 / Total cellular area |
| cc | Area of NDP52 with mt-GFP / Total area of NDP52 |
| dd | Area of NDP52 with mt-GFP / Total area of mt-GFP |
| ee | Area of NDP52 with Proteostat-labeled mt-GFP / Total area of Proteostat-labeled mt-GFP |
| ff | Total area of p62 / Total cellular area |
| gg | Area of p62 with mt-GFP / Total area of NDP52 |
| hh | Area of p62 with mt-GFP / Total area of mt-GFP |
| ii | Area of p62 with Proteostat-labeled mt-GFP / Total area of Proteostat-labeled mt-GFP |
