# Supplementary material for: Mitochondrial dysfunction generates aggregates that resist lysosomal degradation in human breast cancer cells
Source: Cell Death Dis. 2020 Jun 15;11(6):460. doi: 10.1038/s41419-020-2658-y (PMC7296005; doi:10.1038/s41419-020-2658-y)
Supplement: Supplementary file 17 — Supplemental Table 9 [file 41419_2020_2658_MOESM17_ESM.docx]

**Supplementary Table 8:** Parameters, figure locations, factors, degrees of freedom, and F and p values for the main results of two-way ANOVA analyses.

| **Two-Way ANOVA Analyses** | | | | |
| --- | --- | --- | --- | --- |
| Parameter | Figure | Factors | F (DFn, DFd) | p value |
| Aggregate Propensity Factor (APF) | Figure 1A | Interaction | F (12, 60) = 4.235 | P < 0.0001 |
|  |  | Cell type | F (3, 60) = 23.59 | P < 0.0001 |
|  |  | Treatment | F (4, 60) = 50.49 | P < 0.0001 |
| Ubiquitin and Proteostat cell population | Figure 1C | Interaction | F (12, 40) = 63.67 | P < 0.0001 |
|  |  | Quadrant | F (3, 40) = 1060 | P < 0.0001 |
|  |  | Treatment | F (4, 40) = 0.1540 | P = 0.9601 |
| Aggrephagy flux | Supplementary | Interaction | F (7, 80) = 2.371 | P = 0.0296 |
|  | Figure 1C | Treatments | F (7, 80) = 45.99 | P < 0.0001 |
|  |  | Bafilomycin | F (1, 80) = 9.034 | P = 0.0035 |
| GFP-LC3 with or without Proteostat | Figure 1J and | Interaction | F (3, 2792) = 329.3 | P < 0.0001 |
|  | Supplementary | Treatment | F (3, 2792) = 268.4 | P < 0.0001 |
|  | Table 1 | Proteostat | F (1, 2792) = 3139 | P < 0.0001 |
| APF in presence and absence of Bafilomycin | Figure 2D | Interaction | F (15, 96) = 2.935 | P = 0.0007 |
|  |  | Treatment | F (15, 96) = 22.93 | P < 0.0001 |
|  |  | Bafilomycin | F (1, 96) = 45.44 | P < 0.0001 |
| p53 levels | Figure 2I | Interaction | F (3, 16) = 0.2179 | P = 0.8826 |
|  |  | Treatment | F (3, 16) = 2.332 | P = 0.1129 |
|  |  | Bafilomycin | F (1, 16) = 0.1689 | P = 0.6865 |
| p53 levels | Figure 2H | Interaction | F (3, 16) = 1.617 | P = 0.2248 |
|  |  | Treatment | F (3, 16) = 27.60 | P < 0.0001 |
|  |  | Bafilomycin | F (1, 16) = 13.89 | P = 0.0018 |
| Lysotracker staining (Time as a Repeated Measure) | Supplementary | Interaction | F (6, 27) = 80.69 | P < 0.0001 |
|  | Figure 2B | Time | F (3, 27) = 290.4 | P < 0.0001 |
|  |  | Treatment | F (2, 9) = 215.5 | P < 0.0001 |
| Aggregate Propensity Factor (APF) | Supplementary | Interaction | F (2, 12) = 2.413 | P = 0.1315 |
|  | Figure 3C | Treatment | F (2, 12) = 84.22 | P < 0.0001 |
|  |  | ATG7 | F (1, 12) = 17.40 | P = 0.0013 |
| TAX1BP1 levels in MDA-MB-231 cells | Figure 3B | Interaction | F (2, 18) = 1.619 | P = 0.2257 |
|  |  | Treatment | F (2, 18) = 11.46 | P = 0.0006 |
|  |  | Bafilomycin | F (1, 18) = 100.8 | P < 0.0001 |
| TAX1BP1 levels in MCF-12A cells | Figure 3B | Interaction | F (2, 12) = 0.9219 | P = 0.4242 |
|  |  | Treatment | F (2, 12) = 0.5315 | P = 0.6009 |
|  |  | Bafilomycin | F (1, 12) = 82.21 | P < 0.0001 |
| TAX1BP1 levels in MCF-7 cells | Figure 3B | Interaction | F (2, 12) = 3.009 | P = 0.0873 |
|  |  | Treatment | F (2, 12) = 8.672 | P = 0.0047 |
|  |  | Bafilomycin | F (1, 12) = 32.55 | P < 0.0001 |
| TAX1BP1 levels in SKBR3 cells | Figure 3B | Interaction | F (2, 12) = 0.5216 | P = 0.6064 |
|  |  | Treatment | F (2, 12) = 0.3709 | P = 0.6978 |
|  |  | Bafilomycin | F (1, 12) = 59.27 | P < 0.0001 |
| NDP52 levels in MDA-MB-231 cells | Figure 3B | Interaction | F (2, 12) = 23.29 | P < 0.0001 |
|  |  | Treatment | F (2, 12) = 140.8 | P < 0.0001 |
|  |  | Bafilomycin | F (1, 12) = 430.8 | P < 0.0001 |
| NDP52 levels in MCF-12A cells | Figure 3B | Interaction | F (2, 12) = 0.9310 | P = 0.4209 |
|  |  | Treatment | F (2, 12) = 1.830 | P = 0.2024 |
|  |  | Bafilomycin | F (1, 12) = 34.33 | P < 0.0001 |
| NDP52 levels in MCF-7 cells | Figure 3B | Interaction | F (2, 12) = 0.1071 | P = 0.8992 |
|  |  | Treatment | F (2, 12) = 2.296 | P = 0.1431 |
|  |  | Bafilomycin | F (1, 12) = 17.77 | P = 0.0012 |
| NDP52 levels in SKBR3 cells | Figure 3B | Interaction | F (2, 12) = 0.1779 | P = 0.8392 |
|  |  | Treatment | F (2, 12) = 0.4356 | P = 0.6567 |
|  |  | Bafilomycin | F (1, 12) = 25.52 | P = 0.0003 |
| TAX1BP1 and NDP52 Levels | Figure 4A | Interaction | F (1, 12) = 69.48 | P < 0.0001 |
|  |  | SiRNA | F (1, 12) = 69.48 | P < 0.0001 |
|  |  | Protein | F (1, 12) = 0.4509 | P = 0.5146 |
| TAX1BP1 Levels | Figure 4B | Interaction | F (2, 12) = 0.1388 | P = 0.8718 |
|  |  | Treatment | F (2, 12) = 4.454 | P = 0.0357 |
|  |  | Knockdown | F (1, 12) = 31.46 | P = 0.0001 |
| NDP52 levels | Figure 4B | Interaction | F (2, 18) = 3.508 | P = 0.0517 |
|  |  | Treatment | F (2, 18) = 19.88 | P < 0.0001 |
|  |  | Knockdown | F (1, 18) = 28.41 | P < 0.0001 |
| TAX1BP1 levels | Figure 4C | Interaction | F (1, 12) = 0.9476 | P = 0.3495 |
|  |  | Treatment | F (1, 12) = 112.4 | P < 0.0001 |
|  |  | Knockdown | F (1, 12) = 181.8 | P < 0.0001 |
| NDP52 levels | Figure 4C | Interaction | F (1, 12) = 8.912 | P = 0.0114 |
|  |  | Treatment | F (1, 12) = 13.69 | P = 0.0030 |
|  |  | Knockdown | F (1, 12) = 14.00 | P = 0.0028 |
| LC3-II levels | Figure 4C | Interaction | F (3, 32) = 5.379 | P = 0.0041 |
|  |  | Treatment | F (3, 32) = 19.55 | P < 0.0001 |
|  |  | Knockdown | F (1, 32) = 117.6 | P < 0.0001 |
| TAX1BP1 Levels | Figure 4E | Interaction | F (2, 18) = 0.7774 | P = 0.4744 |
|  |  | Treatment | F (2, 18) = 7.919 | P = 0.0034 |
|  |  | Knockdown | F (1, 18) = 17.31 | P = 0.0006 |
| NDP52 levels | Figure 4D | Interaction | F (2, 18) = 0.4120 | P = 0.6684 |
|  |  | Treatment | F (2, 18) = 6.692 | P = 0.0067 |
|  |  | Knockdown | F (1, 18) = 16.78 | P = 0.0007 |
| LC3-II levels (MitoQ Treatment) | Figure 4F | Interaction | F (3, 32) = 0.7743 | P = 0.5170 |
|  |  | Treatment | F (3, 32) = 4.922 | P = 0.0064 |
|  |  | Knockdown | F (1, 32) = 23.64 | P < 0.0001 |
| LC3-II levels (MitoApo Treatment) | Figure 4 F | Interaction | F (3, 32) = 4.477 | P = 0.0098 |
|  |  | Baf | F (3, 32) = 14.77 | P < 0.0001 |
|  |  | Knockdown | F (1, 32) = 93.98 | P < 0.0001 |
| Aggregates in knockdowns | Figure 5A | Interaction | F (9, 57) = 1.798 | P = 0.0885 |
|  |  | Treatment | F (3, 57) = 31.90 | P < 0.0001 |
|  |  | Knockdown | F (3, 57) = 5.009 | P = 0.0038 |
| Mitochondrial delivery to lysosome | Figure 5B | Interaction | F (3, 16) = 1.773 | P = 0.1928 |
|  |  | Knockdown | F (3, 16) = 3.788 | P = 0.0316 |
|  |  | Bafilomycin | F (1, 16) = 34.30 | P < 0.0001 |
| Mitochondrial delivery to lysosome | Figure 5C | Interaction | F (3, 16) = 0.3775 | P = 0.7705 |
|  |  | Knockdown | F (3, 16) = 4.442 | P = 0.0188 |
|  |  | Bafilomycin | F (1, 16) = 486.5 | P < 0.0001 |
| Mitochondrial delivery to lysosome | Figure 5D | Interaction | F (3, 16) = 0.4192 | P = 0.7417 |
|  |  | Knockdown | F (3, 16) = 1.310 | P = 0.3056 |
|  |  | Bafilomycin | F (1, 16) = 110.6 | P < 0.0001 |
| Cell Death | Figure 5E | Interaction | F (9, 32) = 0.8519 | P = 0.5757 |
|  |  | Knockdown | F (3, 32) = 15.67 | P < 0.0001 |
|  |  | Treatment | F (3, 32) = 66.35 | P < 0.0001 |
