# Supplementary material for: Mitochondrial dysfunction generates aggregates that resist lysosomal degradation in human breast cancer cells
Source: Cell Death Dis. 2020 Jun 15;11(6):460. doi: 10.1038/s41419-020-2658-y (PMC7296005; doi:10.1038/s41419-020-2658-y)
Supplement: Supplementary file 18 — Supplemental Table 10 [file 41419_2020_2658_MOESM18_ESM.docx]

**Supplementary Table 9:** Parameters, figure locations, factors, degrees of freedom, and F and p values for the main results of one-way ANOVA analyses.

| **Ordinary ANOVA Analyses** | | | |
| --- | --- | --- | --- |
| Parameter | Figure location | F (DFn, DFd) | p value |
| 1 mM> x < 5 mM Particles in Cell lysate | Figure 1E | F (4, 30) = 5.984 | P = 0.0012 |
| Pearson Correlation GFP-LC3 and Proteostat Punctae | Figure 2a and Supplementary Table 1 | F (3, 9) = 23.80 | P = 0.0001 |
| Population GFP-LC3 and Proteostat Punctae | Figure 2a and Supplementary Table 1 | F (3, 25) = 70.88 | P < 0.001 |
| GFP-LC3 Proteostat Punctae | Figure 1G and Supplementary Table 1 | F (3, 9) = 14.56 | P = 0.0008 |
| Proteostat Punctae | Figure 1G and Supplementary Table 1 | F (3, 9) = 25.87 | P < 0.0001 |
| GFP-LC3 colocalized with Proteostat Punctae | Figure 1I and Supplementary Table 1 | F (3, 9) = 18.64 | P = 0.0003 |
| Proteostat not localized with GFP-LC3 (Area) | Supplementary Table 1 | F (3, 9) = 1.955 | P = 0.1914 |
| Cell Size (Area) | Supplementary Table 1 | F (3, 9) = 8.541 | P = 0.0053 |
| GFP-LC3 Punctae (Area) | Figure 1H and Supplementary Table 1 | F (3, 9) = 9.435 | P = 0.0039 |
| Proteostat Punctae (Area) | Figure 1H and Supplementary Table 1 | F (3, 9) = 6.590 | P = 0.0119 |
| LAMP1- and Proteostat- stained total cell area | Supplementary Table 2 | F (3, 25) = 4.571 | P = 0.0110 |
| Pearson Correlation Lamp1 and Proteostat Punctae | Figure 2A and Supplementary Table 2 | F (3, 9) = 18.90 | P = 0.0003 |
| Cell population with Proteostat punctae with LAMP1 | Figure 2A and Supplementary Table 2 | F (3, 21) = 39.94 | P < 0.0001 |
| LAMP1/Total Cell Area | Figure 2B and Supplementary Table 2 | F (3, 24) = 6.713 | P = 0.0019 |
| Proteostat/Total Cell Area | Figure 2B and Supplementary Table 2 | F (3, 24) = 17.30 | P < 0.0001 |
| LAMP1 Area with Proteostat | Figure 2C | F (3, 24) = 77.19 | P < 0.0001 |
| LAMP1 Levels | Figure 2F | F (4, 20) = 13.55 | P < 0.0001 |
| Cathepsin Levels | Figure 2F | F (4, 10) = 12.56 | P = 0.0007 |
| LAL Levels | Figure 2F | F (4, 25) = 36.69 | P < 0.0001 |
| % of Population positive for p53 and Proteostat colocalization | Figure 2G and Supplementary Table 4 | F (3, 17) = 59.51 | P < 0.0001 |
| Pearson Correlation for p53 and Proteostat | Figure 2G and Supplementary Table 3 | F (3, 8) = 45.62 | P < 0.0001 |
| p53 and Proteostat Punctae per cell | Supplementary Table 3 | F (3, 17) = 51.65 | P < 0.0001 |
| Area of Proteostat Punctae per cell | Supplementary Table 3 | F (3, 17) = 85.08 | P < 0.0001 |
| Proteostat in p53 Area | Supplementary Table 3 | F (3, 17) = 43.75 | P < 0.0001 |
| Protein stability - OPTN Levels at 24 hours | Supplementary Figure 3A | F (3, 12) = 7.124 | P = 0.0053 |
| Protein stability - TAX1BP1 Levels at 24 hours | Supplementary Figure 3A | F (3, 8) = 98.23 | P < 0.0001 |
| Protein stability - p62 Levels at 24 hours | Supplementary Figure 3A | F (3, 12) = 3.373 | P = 0.0546 |
| Protein stability - NDP52 Levels at 24 hours | Supplementary Figure 3A | F (3, 8) = 52.72 | P < 0.0001 |
| Degradation mechanisms - OPTN Levels at 24 hours | Figure 3A - MitoQ | F (3, 12) = 22.25 | P < 0.0001 |
| Degradation mechanisms - TAX1BP1 Levels at 24 hours | Figure 3A - MitoQ | F (3, 16) = 11.41 | P = 0.0003 |
| Degradation mechanisms - p62 Levels at 24 hours | Figure 3A - MitoQ | F (3, 16) = 2.938 | P = 0.0650 |
| Degradation mechanisms - NDP52 Levels at 24 hours | Figure 3A - MitoQ | F (3, 11) = 57.63 | P < 0.0001 |
| Degradation mechanisms - OPTN Levels at 24 hours | Figure 3A - MitoApo | F (3, 8) = 13.99 | P = 0.0015 |
| Degradation mechanisms - TAX1BP1 Levels at 24 hours | Figure 3A - MitoApo | F (3, 8) = 38.00 | P < 0.0001 |
| Degradation mechanisms - p62 Levels at 24 hours | Figure 3A - MitoApo | F (3, 12) = 1.626 | P = 0.2354 |
| Degradation mechanisms - NDP52 Levels at 24 hours | Figure 3A - MitoApo | F (3, 8) = 49.81 | P < 0.0001 |
| Pearson Correlation for mt-GFP and Proteostat in whole cells - p62 stained cells | Supplementary Figure 4D and Supplementary Table 4 | F (3, 17) = 72.99 | P < 0.0001 |
| Pearson Correlation for mt-GFP and Proteostat in whole cells -TAX1BP1 stained cells | Figure 3C and Supplementary Table 5 | F (3, 15) = 32.14 | P < 0.0001 |
| Pearson Correlation for mt-GFP and Proteostat in whole cells - NDP52 stained cells | Figure 3D and Supplementary Table 6 | F (3, 17) = 24.39 | P < 0.0001 |
| Pearson Correlation for mt-GFP and p62 in whole cells - p62 stained cells | Supplementary Figure 4D and Supplementary Table 4 | F (3, 17) = 4.213 | P = 0.0212 |
| Pearson Correlation for mt-GFP and TAX1BP1 in whole cells -TAX1BP1 stained cells | Figure 3C and Supplementary Table 5 | F (3, 15) = 1.100 | P = 0.3798 |
| Pearson Correlation for mt-GFP and NDP52 in whole cells - NDP52 stained cells | Figure 3D and Supplementary Table 6 | F (3, 17) = 5.098 | P = 0.0107 |
| Pearson Correlation for Proteostat and p62 in whole cells - p62 stained cells | Supplementary Figure 4D and Supplementary Table 4 | F (3, 17) = 1.047 | P = 0.3972 |
| Pearson Correlation for Proteostat and TAX1BP1 in whole cells -TAX1BP1 stained cells | Figure 3C and Supplementary Table 5 | F (3, 15) = 0.3006 | P = 0.8244 |
| Pearson Correlation for Proteostat and NDP52 in whole cells - NDP52 stained cells | Figure 3D and Supplementary Table 6 | F (3, 17) = 11.25 | P = 0.0003 |
| Pearson Correlation for Proteostat-labeled mt-GFP and p62 in whole cells - p62 stained cells | Supplementary Figure 4D and Supplementary Table 4 | F (3, 17) = 12.12 | P = 0.0002 |
| Pearson Correlation for Proteostat-labeled mt-GFP and TAX1BP1 in whole cells -TAX1BP1 stained cells | Figure 3C and Supplementary Table 5 | F (3, 15) = 100.7 | P < 0.0001 |
| Pearson Correlation for Proteostat-labeled mt-GFP and NDP52 in whole cells - NDP52 stained cells | Figure 3D and Supplementary Table 6 | F (3, 17) = 1.050 | P = 0.3959 |
| % of area of Proteostat Punctae - p62 stained cells | Supplementary Table 4 | F (3, 17) = 114.2 | P < 0.0001 |
| % of area of Proteostat Punctae - TAX1BP1 stained cells | Supplementary Table 5 | F (3, 17) = 61.85 | P < 0.0001 |
| % of area of Proteostat Punctae - NDP52 stained cells | Supplementary Table 6 | F (3, 16) = 39.18 | P < 0.0001 |
| Percentage of mt-GFP area with overlapping p62 | Supplementary Table 4 | F (3, 17) = 9.390 | P = 0.0007 |
| Percentage of mt-GFP area with overlapping TAX1BP1 | Supplementary Table 5 | F (3, 16) = 9.599 | P = 0.0007 |
| Percentage of mt-GFP area with overlapping NDP52 | Supplementary Table 6 | F (3, 17) = 1.050 | P = 0.3959 |
| Percentage of p62 area with overlapping Proteostat-labeled mt-GFP | Supplementary Table 4 | F (3, 17) = 11.71 | P = 0.0002 |
| Percentage of TAX1BP1 area with overlapping Proteostat-labeled mt-GFP | Supplementary Table 5 | F (3, 15) = 5.013 | P = 0.0133 |
| Percentage of NDP52 area with overlapping Proteostat-labeled mt-GFP | Supplementary Table 6 | F (3, 17) = 1.058 | P = 0.3927 |
| Percentage of p62 area with overlapping Proteostat | Supplementary Table 4 | F (3, 17) = 12.12 | P = 0.0002 |
| Percentage of TAX1BP1 area with overlapping Proteostat | Supplementary Table 5 | F (3, 15) = 100.7 | P < 0.0001 |
| Percentage of MDP52 area with overlapping Proteostat | Supplementary Table 6 | F (3, 17) = 21.92 | P < 0.0001 |
